# Advanced hydrogel material for colorectal cancer treatment

**DOI:** 10.1080/10717544.2024.2446552

**Published:** 2024-12-31

**Authors:** Yu Guo, Min Wang, Yuzhong Zhang, Zeyun Zhao, Jiannan Li

**Affiliations:** a Department of the General Surgery, Jilin University Second Hospital, Changchun, China; b Department of the Gastroenterology, Jilin University Second Hospital, Changchun, China

**Keywords:** Colorectal cancer, hydrogel, cancer, treatment, smart-responsive

## Abstract

Colorectal cancer is one of the most common cancers worldwide, and its incidence rates are increasing every year. Treatments for CRC include surgical resection, chemotherapy, radiotherapy, targeted therapy, and immunotherapy. Although various agents have been used in the treatment of malignant tumors, they are not as effective as expected. This is primarily owing to the lack of selectivity, poor solubility, and severe side effects of most agents. It is necessary to develop more efficient drug delivery systems for the precise targeting of the tumor site and effective therapeutic effects to meet clinical needs. A hydrogel is a three-dimensional network material composed of crosslinked side chains of hydrophilic or hydrophobic groups and a polymer backbone. Hydrogels possess useful properties including high water content, adjustable physical characteristics, elasticity, flexibility, reversible swelling, and multifunctionality. These properties render them ideal biomaterials with a broad range of applications in biomedicine and bioengineering. In this review, we introduce the pathophysiology and current therapeutic advances in CRC and summarize the applications of hydrogels composed of different materials as well as smart response hydrogels as drug carriers in CRC treatment. We also analyze the unique advantages and challenges of using hydrogels as targeted drug delivery carriers in tumor therapy.

## Graphical abstract

**Figure:**
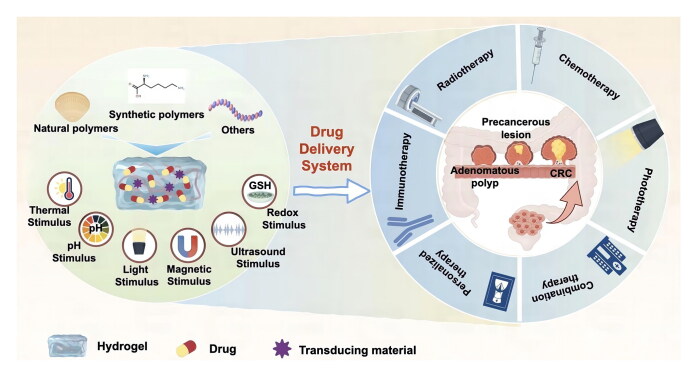

## 1. Introduction

Colorectal cancer (CRC) is one of the most common cancers worldwide, and its incidence rates are increasing every year (Argilés et al., 2020). According to the World Health Organization (WHO), each year, several million individuals are newly diagnosed with CRC, and hundreds of thousands die from it (Deo et al., 2022). The incidence of CRC differs among regions and countries, with a relatively high incidence recorded in North America, possible owing to the high-fat, high-protein diet typical to the region (Xia et al., 2022). Risk factors for CRC include age, diet (high-fat, low-fiber, with a high red meat intake), obesity, physical inactivity, inflammatory bowel disease, and family history (Ngeow & Eng, 2021; Badiani et al., 2022; Gefen et al., 2023). Effective screening and testing can improve the early detection of colonic lesions and increase the remission rate (Pilleron et al., 2021). Common screening methods include fecal occult blood tests, colonoscopy, and abdominal computed tomography (CT).

Treatments for CRC include surgical resection, chemotherapy, radiotherapy, targeted therapy, and immunotherapy. Surgical resection is the primary treatment for early-stage tumors, which involves removing the tumor and adjacent affected tissues (Verkuijl et al., 2021). Chemotherapy plays a significant part in CRC treatment and is commonly prescribed as part of a comprehensive treatment program. First-line drugs for CRC chemotherapy include 5-fluorouracil (5-Fu) and oxaliplatin. However, common chemotherapeutic agents are poorly selective and destroy a large quantity of normal cells while eliminating tumor cells. Typical drug-related side effects include immunosuppression, gastrointestinal reactions, bone marrow hematopoiesis suppression, and sensory abnormalities (Chen et al., 2018). Radiotherapy is a noninvasive, targeted treatment that allows the precise localization of the tumor site. Radiotherapy has a limited range of application, with variable sensitivity of different tissues to radiation, and it can cause damage to normal tissues, causing adverse reactions such as radiation enteritis (Tan et al., 2022; Shi et al., 2023). Furthermore, immunotherapy (Zhao et al., 2022), targeted therapy (Li et al., 2023), photodynamic therapy (PDT) (Yan et al., 2021), and sonodynamic therapy (SDT) (Son et al., 2020), which have advantages such as superior targeting, noninvasiveness, and fewer side effects, have been studied extensively.

Although various agents have been used in the treatment of malignant tumors, they are not as effective as expected. This is primarily owing to the lack of selectivity, poor solubility, and severe side effects of most agents. It is necessary to develop more efficient drug delivery systems (DDS) for the precise targeting of the tumor site and effective therapeutic effects to meet clinical needs. With increasing research on nanomaterials, several drug carriers that can help reduce side effects and enhance the therapeutic efficacy of drugs are being developed. Nanocarriers, such as nanoparticles, quantum dots, organic micelles, and dendritic polymers, are promising agents to improve the shortcomings of various therapeutic strategies against tumors (Cheng et al., 2021). However, most nanocarriers have their limitations and cannot be adapted effectively to therapeutic requirements. The size and structure of quantum dots restrict the drug volume (Dhas et al., 2022). Inorganic materials usually exhibit poor degradability (Pei et al., 2023). Organic micelles are mostly unsuitable for stable drugs release and are poorly biocompatible (Mukhopadhyay et al., 2021), whereas dendrimers exhibit severely limited drug loading capacity (Zhan et al., 2023).

A hydrogel is a three-dimensional network material composed of crosslinked side chains of hydrophilic or hydrophobic groups and a polymer backbone. Hydrogels have useful characteristics, such as a high-water content, tunable physical properties, elasticity, flexibility, reversible swelling, and multifunctionality, which make them ideal biomaterials with a wide range of applications in biomedicine and bioengineering (Mikhail et al., 2023). As a material with a three-dimensional network structure, hydrogels have garnered the attention of researchers studying DDSs. Hydrogels can load drugs on their interior or surface and release drugs stably through adsorption, chemical binding, physical mixing, and membrane embedding. Compared to normal tissues, tumor tissues have specific microenvironments, such as hypoxia and low pH (Guo et al., 2022). The unique porous structure of hydrogels helps water molecules and ions diffuse freely and makes them responsive to external stimuli. This helps form smart-responsive hydrogels that can release drugs in response to internal or external stimuli and reduce damage to normal tissues. Ghobashy et al. prepared pH-responsive hydrogels by cross-linking chitosan with polymers of two anions, acrylic acid and (2-acrylamido-2-methylpropanesulfonic acid), and evaluated their ability to release 5-Fu at different pH values (Ghobashy et al., 2021). Hydrogels have several advantages as emerging carriers for antitumor drugs: (a) excellent biocompatibility: hydrogels exhibit excellent biocompatibility and are naturally degraded in the body without triggering significant immune reactions or toxic side effects; (b) large loading capacity: hydrogels can carry a large number of drug molecules, enabling the efficient treatment of tumor tissues and reducing the drug administration frequency; (c) targeting: the targeted release of drugs can be achieved by regulating the structural properties and porosity of hydrogels or introducing specific active chemical groups; (d) controlled release: the chemical composition and structure of hydrogels can be adjusted to achieve slow drug release and directional delivery, which helps maintain the drug concentration in the tumor tissues in an effective therapeutic range.

In this review, we introduce the pathophysiology and current therapeutic advances in CRC and summarize the applications of hydrogels composed of different materials as well as smart response hydrogels as drug carriers in CRC treatment. We also analyze the unique advantages and challenges of using hydrogels as targeted drug delivery carriers in tumor therapy.

## 2. Pathophysiology of CRC

CRC is the second-leading cause of cancer-related deaths in the United States and the fourth-leading cause of cancer-related deaths globally (Deo et al., 2022). As a common malignant tumor of the digestive system, CRC is commonly detected in the rectum and sigmoid colon. In some cases, it may be detected in the cecum, ascending colon, descending colon, and transverse colon, in the given order.

Typical clinical manifestations of CRC include altered defecation habits, including traits and frequency. Depending on the site of tumor onset, CRC can also form abdominal masses and cause intestinal obstruction and systemic manifestations such as anemia. For patients presenting with symptoms typical to CRC, the common screening methods include fecal occult blood testing and colonoscopy. Colonoscopy, an invasive test for CRC diagnosis, has a high sensitivity and specificity and allows the direct removal of precursor lesions and early cancers (Vitello et al., 2020; Kanth & Inadomi, 2021). Fecal DNA testing has also been recommended by several authoritative organizations in the United States for the early screening of colorectal tumors in asymptomatic populations (Jayasinghe et al., 2023).

Unlike other types of cancer, CRC is associated with a multitude of risk factors. In addition to age and gender, a family history of CRC, inflammatory bowel disease, smoking, excessive alcohol consumption, high red meat intake, obesity, and diabetes are all notable risk factors CTV (Ngeow & Eng, 2021; Badiani et al., 2022). Among these, family history and inflammatory bowel disease are most strongly associated with CRC. Other risk factors are considerably common and play a critical role in the history of CRC. Increasing evidence shows that smoking cessation, a healthy diet, and regular exercise can help protect against CRC. In addition, vitamin supplementation, hormone replacement therapy, and aspirin and non-steroidal anti-inflammatory drug intake are also considered to be associated with a reduced risk of CRC (Shaw et al., 2018).

CRC occurrence and development are believed to constitute a complex multifactorial and multistage process (Li et al., 2021). Most CRCs are caused by polyps, with the process initiating an abnormal glandular fossa. The accumulation of genetic mutations and epigenetics may inactivate or activate oncogenes, leading to the transformation of normal colorectal epithelial cells to precancerous lesions, which consequently results in CRC. Conventional adenomas (including tubular, tubulovillous, and choriocarcinomas) and colorectal serrated lesions are the two major pre-cancerous lesions of CRC (Shaukat et al., 2020). Choriocapillaris adenomas and sessile serrated lesions (SSLs) are considered most likely to progress into CRC. More than 70% of adenoma formation is accompanied by APC gene mutations, indicating that APC gene mutations are closely associated with precancerous lesions in colorectal cancer. In addition, adenoma-cancer progression is often accompanied by KRAS gene activation and P53 oncogene suppression (He et al., 2018; Sninsky et al., 2022). SSL, as a typical precancerous lesion, tends to be characterized by the methylation of the CpG locus and mutations in the BRAF gene (Thorlacius et al., 2017; Mezzapesa et al., 2022). Therefore, the typical molecular characteristics of colorectal cancer include genomic instability, epigenetic abnormalities, and gene expression disorders, along with the high heterogenity of tumor tissues. As a result, patients with CRC suffer from poor prognosis and insensitivity to multiple therapies.

The prognosis of patients with CRC is closely related to tumor staging. Commonly adopted staging systems include the TNM and Dukes staging systems, in which CRC is classified into different stages according to the tumor size, lymph node involvement, and distant metastasis (Banias et al., 2022). The five-year survival rate of patients with stage I tumors is approximately 80%–90%, whereas that of patients with stage IV CRC involving other organs or sites is only 10% (Kc et al., 2023). Distant organ metastasis is one of the leading causes of death in patients with advanced CRC, and the common sites of metastasis include the liver, lung, bone, brain, and peritoneum. It is a tremendous challenge to treat CRC, and there are several new approaches that have been investigated to improve treatment outcomes and survival rates.

## 3. Hydrogels as promising drug carriers for CRC treatment

Hydrogels have unique advantages in helping treat CRC. As a material with a three-dimensional network structure, hydrogels can load drugs on their interior or surface and release drugs stably through adsorption, chemical bonding, physical mixing, and membrane embedding (Li et al., 2023). As a DDS, hydrogels can be naturally degraded in the body without causing obvious immune responses or toxic side effects. The porous structure of hydrogels can carry a large number of drug molecules, thereby realizing efficient treatment of tumor tissues, slow release and targeted delivery of drugs, and helping to maintain the drug concentration in tumor tissues within an effective therapeutic range. At the same time, the unique porous structure of hydrogels helps water molecules and ions diffuse freely, enabling them to respond to external stimuli. This helps form smart responsive hydrogels that can release drugs according to internal or external stimuli and reduce damage to normal tissues.

Smart responsive hydrogels can respond to specific internal or external stimuli, such as changes in temperature, pH value, light irradiation, or enzymatic activity. This enables targeted and controlled release of drugs at the tumor site. For example, thermosensitive hydrogels can release drugs in response to an increase in temperature at the tumor location, while pH-sensitive hydrogels can release drugs in an acidic tumor microenvironment. Yang et al. prepared a thermosensitive methylcellulose injectable hydrogel carrying OXA, which can be used for the treatment of peritoneal metastasis of CRC and showed a significant antitumor therapeutic effect in the CT-26 tumor model (Yang et al., 2024). Sheng et al. prepared a pH-responsive dual-drug-loaded hydrogel based on the changes in pH values at various parts of the gastrointestinal tract (GIT). The alginate and sodium carboxymethyl cellulose hydrogel can protect methotrexate and aspirin from being absorbed by the stomach and small intestine (Sheng et al., 2021). Secondly, the porous structure of hydrogels allows for the extensive encapsulation and slow release of drugs, which can maintain an effective drug concentration at the tumor site for a long time and enhance the therapeutic effect. Furthermore, hydrogels can be functionalized with ligands or antibodies that specifically target tumor cells, thereby improving the specificity and effectiveness of drug delivery. Hydrogels can release anticancer drugs directly to the tumor site, exerting cytotoxic effects on tumor cells. They can also enhance the immune response against tumors by modulating the tumor microenvironment. In general, hydrogels offer a promising approach for the treatment of CRC with their unique characteristics and ability to achieve controlled drug release and tumor cell killing.

The therapeutic efficacy of hydrogel-loaded drugs to kill CRC has been investigated in both *in vitro* and *in vivo* experiments and clinical trials. The commonly researched CRC cell lines were human-derived HCT116, SW480, SW620, and Caco-2, and murine-derived CT26. Md et al. used a protein-polysaccharide complex water-based gel to load naringin, a natural anticancer component with poor water solubility, which greatly increased the solubility of naringin and showed a significant killing effect on the HCT116 cell line (Md et al., 2022). The carboxymethyl tapioca starch/arginine hydrogel rich in MgFe_2_O_4_ nanoparticles with dual pH and magnetic responsiveness prepared by Fang et al. and loaded with DOX also exhibited obvious cytotoxicity to the CRC cell line HCT116 (Fang et al., 2022). Short interfering RNA technology is an emerging option for the effective treatment of CRC. Liechty et al. performed a pH-responsive hydrogel for loading functional siRNAs and demonstrated that the investigated nanogel has the ability to deliver siRNAs and induce gene knockdown with the human CRC cell line, Caco-2 (Liechty et al., 2019).

Animal models frequently selected to validate the efficacy of hydrogel-loaded therapy for CRC are the HCT116 subcutaneous tumor model, the orthotopic rectal cancer model, and the CT26 peritoneal carcinomatosis mice model. Hypothermia employs a relatively low temperature (<45 °C) to destroy cancer cells, while at the same time is less toxic to normal tissues. Ouyang et al. prepared an in situ hydrogel of sodium alginate, encapsulated with an ink photothermite and an azo initiator, 2,2′-azobis[2-(2-imidazolidin-2-yl)propane] dihydrochloride, which was irradiated by a NIR II laser. A significant tumor growth inhibitory effect on the HCT116 subcutaneous CRC graft tumor model was observed (Ouyang et al., 2019). The mouse orthotopic CRC model is an important model for evaluating the treatment, distribution, and safety of drug-loaded hydrogels. Cao et al. used chitosan/alginate oral hydrogels loaded with ultrasound-driven nanomotors and immune checkpoint inhibitors to inhibit tumor growth in the CT26 orthotopic mouse model by directly killing and enhancing systemic antitumor immunity (Cao et al., 2022). CRC-complicated peritoneal metastases serve as a major challenge for clinical treatment, and the stronger tissue adhesion of injectable hydrogels can effectively overcome the limitation of high clearance of intraperitoneal chemotherapy. Wang et al. designed a hydrogel delivery system of PLEL loaded with OXA and raquinimod, which was injected into the peritoneal cavity of mice, and was shown to be effective in treating tumors, inhibiting the production of ascites, and significantly prolonging the lifespan of mice in the peritoneal metastasis mouse model of CT26 (Wang et al., 2023).

As a drug carrier with great potential, some drug-loaded hydrogels have entered the clinical trial stage of CRC treatment. We searched for clinical trial information registered in Clinical Trials (https://clinicaltrials.gov/) and the International Clinical Trials Registry Platform (ICTRP Search Portal (who.int)) and found that a total of four drug-loaded hydrogels have entered the clinical trial stage and are recruiting patients. Zhang et al. intend to utilize colonoscopic bowel for flexible coverage of the entire colon and enhanced 5-Fu adhesion locally with a thermosensitive hydrogel (NCT 06385418). Thomas et al. focused on the feasibility and efficacy of TraceIT^®^, an injectable hydrogel spacer, for vaginal/prostate preservation during radiotherapy in rectal cancer patients (NCT03258541). Spanish researchers employed LIFEPEARLS^®^ to form composite irinotecan into hydrogel microspheres to help irinotecan penetrate more deeply into tumor tissue for the treatment of CRC and mCRC (NCT04595266). Similar clinical studies have explored the feasibility and tolerability of combining thermosensitive hydrogels loaded with two immunomodulators (GMCSF and mifamurtide) for the treatment of unresectable CRC liver metastases (NCT04062721).

As an emerging drug carrier with superior biocompatibility, hydrogels can achieve controlled and targeted drug release, reduce drug-induced adverse effects, and enhance the therapeutic efficacy of drugs. These properties make hydrogels effective treatment agents for CRC.

## 4. Biomaterials used to prepare hydrogels for CRC treatment

As a new type of DDS, hydrogels have advantages such as favorable biocompatibility, biodegradability, efficient drug loading, and controlled drug release. Hence, they are widely used in cancer chemotherapy, radiotherapy, immunotherapy, and PDT. Based on the source of the material, hydrogels can be categorized as natural hydrogels, synthetic hydrogels, and composite hydrogels. Most natural hydrogels have excellent biocompatibility and biodegradability and are less susceptible to allergic or rejection reactions, but exhibit poor performance stability. Conversely, synthesized hydrogels exhibit stable properties and have a low preparation cost and superior controllability. However, they may cause irritation or may be prone to rejection.

### 4.1. Natural hydrogel materials

Natural hydrogels are extensively used as drug carriers in tumor therapy. They can effectively improve the stability of drugs, control the release rate of drugs, and enhance drug accumulation in tumor tissues. Because they have a natural origin, they exhibit superior biocompatibility and biodegradability, which reduces their side effects and biotoxicity.

Chitosan is a natural polysaccharide compound composed of N-acetylglucosamine and glucose. Chitosan is available widely in the shells of crustaceans, such as shrimp, crabs, and shellfish, and can also be extracted from bacteria and fungi. As a natural cationic polysaccharide that can be isolated from a broad range of sources, chitosan exhibits several unique biological properties, such as antioxidant, antitumor, anti-inflammatory, antimicrobial, and immunomodulatory properties. Chitosan was found to suppress VEGF and inhibit the formation of tumor vascular endothelial cells, which consequently inhibited the infiltration or metastasis of tumors to adjacent tissues (Wang & Liu, 2014; Wang et al., 2021). Anushree et al. modified chitosan by phosphorylation and galactosylation and synthesized phosphorylated galactosyl chitosan (PGC). PGC exhibited high affinity for ASGPR, a cytosolic glycoprotein receptor present on the surface of hepatocytes, and also showed promising antioxidant and therapeutic potential in hepatocellular carcinoma (U et al., 2022). Moreover, chitosan is soluble in water and can be readily used to prepare solutions, hydrogels, films, fibers, and nanoparticles. Chitosan is a special polymer that dissolves at a low pH, exhibiting pH-responsiveness as a drug carrier (Zhao et al., 2023). Its range of pH sensitivity can be adjusted by incorporating various ionizable functional groups (e.g. carboxyl groups). Ghobashy et al. prepared an amphiphilic hydrogel by cross-linking chitosan and an anionic polymer with two (acrylic acid)-co-(2-acrylic acid-2-methylpropane-sulfonic acid) (AAc/AMPS) using gamma irradiation. This could function as an oral drug carrier to prevent drug release in low pH environments, such as in the presence of gastric juices, for 5-Fu delivery (Ghobashy et al., 2021). Thermosensitive hydrogels can be readily controlled both *in vitro* and *in vivo*. The introduction of β-glycerophosphate as a buffer into chitosan solutions enables a thermosensitive solution-gel transition at body temperature. Zheng et al. combined PDT with chemotherapy for treating localized tumors using thermosensitive chitosan hydrogels delivering the photothermal material MoS_2_ and the chemotherapeutic drug doxorubicin (DOX) (Zheng et al., 2019).

Alginate is a polysaccharide compound derived from marine organisms, such as algae. It meets the safety requirements for drug carriers, including excellent biocompatibility, biodegradability, nonirritating, nontoxic, and non-immunogenic for humans (Zhang & Zhao, 2020; Reig-Vano et al., 2021; Farshidfar et al., 2023). The degradation of alginate does not exert any cytotoxic effects or deleterious effects on genetic information. Alginate with a molecular weight as low as less than 50 kDa can be cleared effectively by the kidneys (Tomić et al., 2023). The solution-gel morphology transition occurs when the negatively charged carboxyl residue of the alginate chain interacts with multivalent cations or cationic polymers, and calcium ions act as the most popular cross-linking agent (Boi et al., 2020; Zhang et al., 2021). Alginate hydrogels serve as drug carriers to protect encapsulated compounds from the environment and enhance their bioavailability. Ji et al. prepared a therapeutic hydrogel platform using the Ca^2+^-mediated cross-linking of Fe_3_O_4_ nanoparticles and alginate hydrogel precursors. The products could aid the efficient absorption of Fe_3_O_4_ nanoparticles by CT26 cancer cells and induce CT26 cell death *in vitro* under near-infrared (NIR) laser irradiation without causing any obvious cytotoxicity (Ji & Wang, 2023). Alginate hydrogels can be easily modified to meet the practical requirements for the loading and delivery of different drugs. Jia et al. enhanced the anticancer effects of DOX by modifying alginate and crosslinking DOX and polyethylene glycolized oxidized alginate nanoparticles with a pH-sensitive Schiff-Base to facilitate pH-responsive DOX delivery in an acidic tumor microenvironment (TME) (Jia et al., 2016). In addition, alginate can interact with glycoproteins in the gastrointestinal tract (GIT) to induce mucosal adhesion, which can prolong the retention of drugs in the GIT. Thus, alginate serves as an ideal oral drug carrier (Sosnik, 2014). Wang et al. developed a colon-targeted oral drug delivery system using two natural polysaccharides: alginate and chitosan. The system was shown to enhance the release of epimedoside in the colon and reduce mucosal damage, which led to a colon-protective effect (Wang et al., 2016). Targeted drug delivery can also be achieved by incorporating ligands in an alginate matrix. Rajpoot et al. showed the improvement of targeted therapeutic efficacy with COLO205 cell and HT29 cell xenografts in mice using solid lipid nanoparticles loaded with folic acid (FA) and irinotecan hydrochloride encapsulated in alginate and Eudragit S100 (Rajpoot & Jain, 2020).

Hyaluronic acid (HA) is an acidic polysaccharide polymer alternately linked by N-acetylglucosamine and glucuronic acid. HA is present extensively in human tissues, such as the skin, cartilage, and vitreous body (Nadra et al., 2021; Saha & Rai, 2021; Unni et al., 2021). Therefore, HA has favorable compatibility with human tissues and is not likely to cause immune reaction or undergo rejection. It can be degraded by physiological enzymes and does not form long-term residues, thereby reducing the potential safety risk. However, pure HA is soluble in water, and its retention time is relatively short, which significantly restricts its wide application. By modifying the hydroxyl, carboxyl, and amide groups in HA through amidation, esterification, ring opening, cross-linking, and compounding, the performance of HA can be improved considerably, which significantly widens the scope of its application in medicine (Pandit et al., 2019; Tiwari & Bahadur, 2019). Wang et al. used 1-(3-dimethylaminopropyl)-3-ethylcarbodiimide hydrochlovide (EDC) and 4-dimethylaminopyridine (DMAP) as esterification activators to link the hydroxyl group of paclitaxel (PTX) with the carboxyl group on the primary chain of HA. They also prepared surface-modified cell-penetrating peptides for antitumor therapy with PTX (Wang et al., 2017). HA, as a non-antigenic molecule, can be compounded with other materials to achieve complementary advantages. Li et al. successfully prepared pH-sensitive composite hydrogels by compounding HA with chitosan (Li et al., 2024). HA hydrogels have unique advantages as DDS for antitumor drugs. HA and its derivatives can bind to specific receptors on the cell surface for targeted drug delivery. CD44 and receptor for hyaluronate-mediated motility receptors (RHAMMs) are overexpressed on the surface of a wide range of tumor cells and can be bound to HA-modified vectors for the tumor-targeted delivery of drugs using the ligand-receptor binding mechanism (Zhang et al., 2016; Wickens et al., 2017; Luo et al., 2019). Fiorica et al. prepared injectable *in situ*-forming hydrogels using HA that can be used for targeted drug release at the tumor site. Evidence from *in vivo* studies has confirmed that drug hydrogels can substantially reduce tumor size in animal tumor models without causing excess cytotoxicity (Fiorica et al., 2020). Peritoneal adhesions and peritoneal metastases following CRC surgery are major clinical challenges. HA hydrogels perform excellently when used to minimize post-surgical peritoneal adhesions. Lee et al. designed and synthesized oxaliplatin-loaded HA and sodium carboxymethylcellulose (CMCNa)-crosslinked hydrogels. This treatment effectively prevented intraperitoneal adhesions in SD rats after CRC surgery (Lee et al., 2019).

Cellulose is a macromolecular polysaccharide that is abundant in nature. It has superior mechanical strength and durability. However, cellulose is difficult to dissolve in water, and its dissolution requires the use of solvent systems, such as NaOH/urea, which severely limits its applications (Zainal et al., 2021). Cellulose derivatives, such as hydroxypropyl methylcellulose (HPMC) (Zhao et al., 2016), hydroxyethyl cellulose (HEC) (Gorgieva & Kokol, 2011), and carboxymethyl cellulose (CMC) (Rasoulzadeh & Namazi, 2017), are widely used in medical research. Among these, the carboxymethyl functional group of CMC aids solution-gel transition under different pH conditions and can be used for the site-specific targeted delivery of drugs. At a low pH, the carboxymethyl group is in the unionized state and the CMC molecule is highly soluble, whereas at a high pH, the carboxymethyl group undergoes ionization and forms a gel structure. Sheng et al. prepared a dual-drug-loading system comprising methotrexate and aspirin using alginate and CMC hydrogels to achieve pH-responsive drug delivery. This could provide both chemotherapeutic and pain relief effects for patients with CRC (Sheng et al., 2021). Yin et al. prepared self-repairing injectable hydrogels using DOX-loaded oxidized CMC and poly(aspartic acid hydrazide) (PAH) and loaded them with the antitumor agent camptothecin (CPT). The synergistic antitumor therapeutic effect of the dual drugs was significantly stronger, and they reduced the side effects of high-dose single drugs (Yin et al., 2023). Cellulose can dissociate laterally in the amorphous region present along its axis to form nanoscale crystals. Vakili et al. developed hydrogels with superior adhesive properties using cellulose nanocrystals (CNC) and polyacrylic acid. These hydrogels could be used for the topical delivery of cisplatin in CRC (Vakili et al., 2021).

Collagen is a complex protein with a triple helical structure. It is characterized by a high degree of stability and mechanical strength. Collagen is widely found in connective tissues, skin, bones, blood vessel walls, and other tissues in living organisms, where it performs vital roles related to support, structural stability, and cell adhesion (Hennet, 2019; Leite et al., 2021). The introduction of collagen biopolymers can effectively improve the mechanical strength of natural hydrogels such as alginate, chitosan, and HA (Moxon et al., 2019). Hwang et al. synthesized an injectable thermos-responsive hydrogel using alginate collagen and evaluated the therapeutic efficacy and anti-metastatic potential of the hydrogel in combination with photothermal therapy (PTT) and immunotherapy against CT-26 cancer in mice (Hwang et al., 2022). Depending on the chemical bonding or physical cross-linking between collagen proteins, a stable gel structure can be formed. Collagen hydrogels are highly porous and have a large surface area, which can provide a favorable environment for cell adhesion and growth and promote cell migration and tissue regeneration (Huang et al., 2023). Collagen hydrogels can be used for the modeling of microenvironments. Devarasetty et al. established a unique CRC organ model using collagen hydrogels to study the effect of matrix topology on the phenotype and proliferation of cancer cells (Devarasetty et al., 2017).

Gelatin is a protein-based biomaterial commonly obtained by fractional hydrolysis or the thermal deformation of collagen extracted from the bones, skin, tendons, and connective tissues of terrestrial animals. Gelatin serves as a natural polymer and is approved by the United States Food and Drug Administration (USFDA) for applications in the biomedical and food industries. It has several advantages, such as accessibility, decomposition, excellent biocompatibility, non-immunogenicity, and ease of preparation and modification (Mohanto et al., 2023). Gelatin hydrogels prepared via physical or chemical cross-linking perform excellently as a promising DDS for the treatment of multiple diseases, including cancers. Akhlaq et al. prepared pH-responsive hydrogels using gelatin and polyvinyl alcohol (PVA) for delivering methotrexate to colonic tissues, wherein different pH values could alter the degree of swelling of the hydrogel to deliver the drug to the colonic site (Akhlaq et al., 2021). Gelatin has superior tissue adhesion properties. Mizuno et al. prepared sealing hydrogels composed of decyl-modified gelatin and polyethylene glycol (PEG)-based cross-linkers to alleviate or prevent tissue adhesions during CRC surgery and anastomotic fistulae after surgery (Mizuno et al., 2020). Similar to those of collagen, the superior adhesion properties of gelatin hydrogels also aid the three-dimensional *in vitro* culture of tumor cells (Pamplona et al., 2024).

The use of natural hydrogels as DDS in tumor treatment has several challenges, such as the uneven rate of biodegradation, which may be affected by the internal environment of the organism, hindering sustained and uniform drug release (Askari et al., 2023). In addition, the structural properties of natural hydrogels may limit efficient drug loading, thus affecting the drug delivery and release efficiency.

### 4.2. Synthesized hydrogel materials

Synthetic hydrogels prepared using physical or chemical methods are highly adjustable and can be used for loading different drugs. Moreover, synthetic hydrogels are easy to modify and have multiple advantageous properties, such as targeted delivery, ease of imaging, and antimicrobial effects, particularly when functional groups or nanomaterials are added to them.

PEG has excellent properties, such as favorable water solubility and biocompatibility, low toxicity and immunogenicity, good stability, and tunable physicochemical properties. These make PEG widely useful and add to its research value in biomedical fields (Fu et al., 2021; Wang et al., 2023). PEG hydrogels can be loaded with drugs via non-covalent capture, and with the progressive cleavage of the reaction segments, the PEG network degrades and releases the drug without forming excess undesirable or toxic monomers (Liu et al., 2022). Zuo et al. illustrated that PEG hydrogels can be almost completely excreted from the body within 10 days of implantation (Zuo et al., 2022). The hydroxyl group at the end of PEG can be readily functionalized and modified to bind to various targeting groups or responsive fragments (e.g. o-ester, imine, ketone, and acetal) to exhibit responsiveness to stimuli such as pH, temperature, and redox changes. This can consequently facilitate drug release in the TME (Yu et al., 2016). PEG hydrogels can be easily modified by disulfide bonds to induce reduction responsiveness (Yan et al., 2010). Wang et al. prepared a pH and reduction dual-responsive smart delivery system loaded with curcumin and DOX using hydrogels as carriers. These carriers could exhibit prolonged drug cycling and improved drug uptake (Lin et al., 2019). Ahmad et al. synthesized an oral hydrogel that can target the colon to release oxaliplatin in response to the free radical polymerization of pH-sensitive methacrylic acid (MAA) and PEG (Barkat et al., 2018). PEG hydrogels can also be used as a collaborative treatment platform for chemotherapy and PTT. Gao et al. incorporated PEG-coated gold nanorods and D-alpha-tocopheryl PEG 1000 succinate (TPGS)-coated paclitaxel nanocrystals into an *in situ* injection hydrogel system. The gold nanorods in this therapeutic platform generated heat to shrink tumors under NIR laser irradiation. This was followed by the sustained release of PTX and the P-glycoprotein inhibitor TPGS to achieve long-term tumor control (Wang et al., 2018). Stimuli-responsive PEG hydrogels are generally associated with poor responsiveness, sensitivity, and precision. Yang et al. prepared a non-responsive conventional PEG hydrogel using metal-free organocatalytic ring-opening polymerization and other methods. This could significantly enhance DOX loading, and the platform improved the tumor-inhibitory effects without inducing significant side effects, such as cardiotoxicity (Yang et al., 2015).

Polycaprolactone (PCL) is a synthetic polyester-based polymer with excellent biocompatibility, biodegradability, and non-immunogenicity. PCL is extensively used in biomedical applications, such as in the preparation of DDS, tissue engineering scaffolds, and medical sutures, among other materials (Siddiqui et al., 2018; Shahverdi et al., 2022). PCL, as a controlled-release material for drugs that degrades gradually in the human body, is currently approved by the USFDA (Azari et al., 2022). Liu et al. prepared a nuclear/shell fiber scaffold drug delivery vehicle by uniformly coating PCL onto DOX-loaded alginate-gelatin hydrogels. PCL coating can effectively reduce the free diffusion of drugs from the gel, increasing the retention of the drugs in the body and facilitating sustained drug release (Liu et al., 2021). Hydrophobic drugs have poor solubility and are susceptible to side effects, such as limited bioavailability and toxin accumulation. Wu et al. encapsulated the strongly hydrophobic antitumor molecule chetomin in a thermosensitive PCL hydrogel, which effectively inhibited tumor growth when applied to a subcutaneous CT26 tumor model. The hydrogel was also observed to inhibit tumor-induced angiogenesis and tumor growth in a transgenic zebrafish model (Wu et al., 2014). Ren et al. used biodegradable thermosensitive PCL hydrogels loaded with oxaliplatin and tannic acid nanoparticles that could significantly limit *in vivo* tumor cell growth and prolong survival in model mice with CT26 peritoneal colon cancer (Ren et al., 2019). PCL also has excellent compatibility with other polymers, and it can be used to prepare various copolymers or blends with superior performance. Consequently, research on PCL and its copolymers and blends as drug carriers has garnered extensive attention (Maspes et al., 2021). Diblock copolymers or triblock copolymers synthesized using PEG and PCL have numerous advantages, including the ease of one-step synthesis, lack of the need for using toxic coupling agents, and longer *in vivo* persistence. Wang et al. used PEG/PCL diblock copolymers for the co-loading of 5-Fu and gene DNA sequences (pEGFP), which considerably enhanced the antitumor effect and gene delivery efficiency (Wang et al., 2018). As a DDS used in tumor therapy, PCL hydrogels exhibit superior controlled drug release, biocompatibility, and biodegradability, which help improve the targeted delivery of drugs.

Polylactic acid (PLA) is a polymer obtained by the polymerization of lactic acid produced by biological fermentation as the primary raw material. It is an ideal green polymer that can be biodegraded, because its raw materials are sufficiently sourced and renewable and its production process is pollution-free. PLA also has reliable biosafety, excellent mechanical properties, and ease of processing and has been reported to be suitable for preparing biodegradable sutures, stents, and DDS (Chen et al., 2023). Nonfunctional PLA is commonly copolymerized with other hydrophilic monomers or conjugated with hydrophilic segments to form hydrogels. For example, PLA can be copolymerized with hydrophilic polymers, such as PEG, to form thermo-responsive hydrogels for the delivery of chemotherapeutic drugs (Movaffagh et al., 2022). PLA has two chiral structures—poly(L-lactic acid) (PLLA) and poly(D-lactic acid) (PDLA)—and is generally prepared by the ring-opening polymerization of propylene glycol esters or the direct condensation of lactic acid. PLLA-PEG-PLLA triblock hydrogels can exhibit sol-gel transition over a wide temperature range when the ratios of the different copolymers are adjusted. They also exhibit a high drug-loading capacity (Liu et al., 2023). PLA can be crosslinked with various polymers to form smart-responsive hydrogels to meet the drug delivery requirements under different conditions. Zhao et al. prepared a thermal/pH dual-responsive hydrogel crosslinked with poly(2-ethyl-2-oxazoline) and PLA for DOX delivery, in which the surface was modified with FA (Zhao et al., 2015). When the drug platform is recognized and internalized by folate receptors on the surface of tumor cells, the drug can be released in lysosomes present in acidic conditions.

Polyglycolic acid (PGA) is prepared by polymerizing α-hydroxy acids and has a simple linear molecular structure. As a natural product synthesized by the human body during metabolism, PGA is biocompatible and biodegradable, which are unique properties and make PGA suitable for biomedical research applications. For example, it is used for preparing medical sutures, DDS, fracture fixation materials, and tissue engineering scaffolds (Su et al., 2021). PGA can be prepared by direct condensation polymerization and ethylenic ester ring-opening polymerization. However, the oligomers prepared by direct condensation polymerization have poor properties, are vulnerable to decomposition, and exhibit weak mechanical strength, whereas the ethyl ester ring-opening polymerization method requires raw materials with a high purity and has a high synthesis cost (Maghsoudi et al., 2020). As green materials with excellent biodegradability, PLA and PGA have significant differences in their degradation rate, mechanical properties, and solubility. Poly(lactic-co-glycolic acid) (PLGA), which is prepared by mixing PLA and PGA, can be modified to control its solubility. The ratio of PLA and PGA can be altered to modify PLGA for specific applications in clinical settings.

PLGA is a biodegradable polymer composed of a copolymer of lactic acid and glycolic acid and exhibits excellent biocompatibility (Mir et al., 2017). PLGA has various attractive and promising properties as a raw material for drug carriers, such as simple preparation methods and the availability of different forms of PLGA that can be prepared by adjusting the ratio of the two monomers. In addition, PLGA is degraded into lactic acid and hydroxyacetic acid, which are also by-products of human metabolic pathways. Therefore, it does not cause any undesirable toxic side effects to the human body when used as a pharmaceutical material, and its use in the parenteral administration of drugs in the pharmaceutical field has been approved by the FDA and the European Medicines Agency (Danhier et al., 2012; Narmani et al., 2023). For example, Lupron Depot, a commercially available treatment for advanced prostate cancer, is a drug carrier based on PLGA (Shirley, 2023). Yu et al. designed a multifunctional hydrogel carrying Fe_3_O_4_ and glucose oxidase (GOx) using a PLGA platform. This could trigger GOx release and inhibit ATP production through the magneto-thermal effect, achieving magneto-thermal therapeutic effects along with the starvation of osteosarcoma cells and the mutual reinforcement of the therapeutic effect (Yu et al., 2023). PLGA can form copolymers with other polymers as well. Ci et al. demonstrated the superior antitumor efficacy of a thermosensitive PLGA-PEG-PLGA copolymer hydrosol loaded with the moderately soluble drug irinotecan injected into an SW620 xenograft tumor model mice (Ci et al., 2014). Piao et al. selected thermosensitive PLGA-PEG-PLGA hydrogels as therapeutic carriers. The EGF-loaded hydrogel could significantly inhibit cervical cancer progression in immunized mice as a slow-release drug carrier by delaying cell cycle progression and decreasing cell proliferation and migration through the sustained release of EGF at low concentrations (Piao et al., 2024).

Polyacrylic acid (PAA) is a linear or crosslinked polymer composed of repeating acrylic monomer units that exhibit significant pH responsiveness. PAA contains several carboxyl structures, which may undergo protonation or deprotonation reactions in different pH environments, alter the overall charged nature of the PAA molecule, and exhibit pH responsiveness. The strong adhesion of PAA to tissues facilitates the local delivery of drugs, which has a broad application prospect in biomedical fields. Li et al. prepared nanogels with calcium carbonate nanoparticles and PAA using ultrasound effects. These nanogels exhibited good stability and excellent DOX-loading ability (Li et al., 2017). The free carboxyl groups of acrylic acid can be applied to load drug molecules by electrostatic adsorption. Wu et al. designed stimuli-responsive hydrogels using PAA as a co-administration system for adriamycin and cisplatin that demonstrated efficient drug loading and delivery (Wu et al., 2017). The anionic acrylic monomer can also be bound to the cationic polysaccharide chitosan to form a stable structure with amphiphilic characteristics of polyionic complex phase stabilization. Abdouss et al. synthesized pH-sensitive chitosan/PAA hydrogels for curcumin delivery by water/oil/water emulsification. These hydrogels exhibited excellent pH-sensitive drug release profiles and antitumor potential (Abdouss et al., 2023). Amini et al. loaded 5-Fu into a magnetic nanocomposite hydrogel composed of chitosan/PAA/Fe_3_O_4_, which aided controlled drug release under both colonic and rectal conditions (Amini-Fazl et al., 2019). Poly(methacrylic acid) (PMAA), a derivative of PAA, is also available for drug carrier studies. Kozlovskaya et al. prepared a novel hydrogel cube exhibiting pH-triggered DOX release through the sequential infiltration of PMAA and poly (N-vinylpyrrolidone) (Kozlovskaya et al., 2014). The steric hydrogel showed rapid and reversible volume changes in response to changes in the solution pH. Pan et al. prepared PMAA-based nanohydrogels with redox and pH dual-stimulation responses that were capable of accelerating DOX release at a lower pH and in a reducing TME (Pan et al., 2012).

Self-assembled peptides (SAPs) are peptide molecules with specific amino acid sequences that spontaneously form ordered biocompatible and sensitively responsive two- or three-dimensional structures via non-covalent interactions (e.g. hydrogen bonding, hydrophobic interactions, π-π stacking, and others) (Zou et al., 2020). SAP can carry various active functional groups (e.g. carboxylic acid, hydroxyl, amino, and thiol groups) on its side chain, which can be chemically modified to enable the functions of DDS (Eskandari et al., 2017; Yang et al., 2021; Zhou et al., 2023). Zhong et al. designed and prepared an octapeptide (FHFDFHFD) hydrogel for the delivery of the anticancer drug tanshinone, which exhibited superior drug loading capacity, sustained drug release, and favorable anticancer potential (Yin et al., 2017). Ghosh et al. used a cyclic dipeptide (CDP) cyclo-(Leu-S-Bzl-Cys) hydrogel loaded with 5-Fu that exhibited better antitumor activity than 5-Fu administered alone in a human CRC cell line HCT116, revealing its potential application in sustainable drug delivery (Ghosh et al., 2022). Peptides with targeted properties can be used for the targeted delivery of drugs. Wang et al. prepared an *in situ*-forming hydrogel by coupling supramolecular nanotube components formed by the tumor-penetrating peptides iRGD and coptisine with a negatively charged stimulator of interferon genes (STING) agonist. This induced tumor regression, increased animal survival, and protected mice against tumor recurrence and metastasis (Wang et al., 2020). The flexible responsiveness of SAP to local microenvironmental stimuli, such as pH, temperature, ionic strength, and enzyme catalysis, in lesions has garnered attention in drug delivery research in oncology (Li et al., 2023). The asparagine in RADA-F6 peptide can undergo negatively charged ionization and positively charged deionization reactions at different pH values. This results in different hydration and hydrophilicity patterns of the RADA peptide at different pH values, which alters its self-assembling behavior and gelation ability (Wang et al., 2019). Ashwanikumar et al. demonstrated that SAP nanohydrogels based on the RADA-F6 peptide could carry 5-Fu and enable its sustained release at an alkaline pH for the efficient treatment of CRC (Ashwanikumar et al., 2016). Poly-lysine, as a cationic peptide, is readily attracted to anionic drugs to form stable complexes. Wu et al. prepared *in situ*-forming hydrogels of poly-lysine loaded with metformin and 5-Fu using tetra-armed polyethylene glycol (PFA) as a cross-linking agent. These hydrogels exhibited a superior therapeutic effect by inhibiting CRC progression in tumor-bearing mice inoculated with C26 (Wu et al., 2016). SAP hydrogels possess unique advantages in drug delivery owing to their facile synthesis, excellent gelling ability, and biocompatibility. However, the otherwise stable SAP-loaded drug is susceptible to external environmental stimuli, such as pH and temperature, and peptides with a large number of amino acids are at potential risk of inducing heightened immune responses.

PVA serves as a nonionic hydrophilic polymer with superior biocompatibility and biodegradability. PVA is currently well represented in biomedical applications, such as in hemostatic dressings, as well as in contact lenses, orthopedic devices, and DDS. The use of physical methods to prepare PVA hydrogels, such as repeated freezing and thawing, helps prevent the introduction of toxic chemical cross-linking agents and makes PVA hydrogels suitable DDS materials. The three-dimensional network structure of PVA hydrogels enhances their drug loading capacity, aiding the encapsulation and controlled release of antitumor drugs (Wu et al., 2023). Hakimi et al. used chitosan/PVA hydrogels loaded with amygdalin, a natural antitumor drug, in a study. The hydrogels exhibited superior biodegradability and biocompatibility while effectively inducing apoptosis in an SW-480 cell line (Hakimi et al., 2023). With simple modifications and alterations, PVA hydrogels can serve as materials responsive to specific stimuli. pH-responsive hydrogels prepared using PVA/gelatin effectively aided the delivery of methotrexate in the colon (Akhlaq et al., 2021). A chemically cross-linked hydrogel composed of PVA/carboxymethyl chitosan prepared by free radical polymerization exhibited pH responsiveness and aided the oral administration of oxaliplatin. This hydrogel caused no adverse drug reactions, and its use reduced the frequency of administration and improved patient compliance (Ullah et al., 2019). The use of PVA hydrogels in tumor treatment has multiple advantages, including greater drug efficacy, fewer toxic side effects, and greater success in precision therapy, making PVA a promising material for tumor treatment.

In composite hydrogels formed by combining multiple polymers either physically (through co-precipitation and electrostatic spinning) or chemically (via copolymerization, grafting, and click chemistry), the properties of the component polymers are combined to obtain improved physical and biological properties or specific functionalities that respond better to the requirements of tumor therapeutic drugs used for delivery (Choi et al., 2023). PLGA/PEG/PLGA triblock hydrogels prepared using ring opening polymerization, which exhibit temperature-responsive solution-gel transition, transform to the gel state, and release drugs at body temperature, have been studied in various tumor therapeutic applications (Norouzi et al., 2021; Kim et al., 2023). For example, Yang et al. demonstrated that PLGA/PEG/PLGA hydrogels loaded with DOX and curcumin show better antitumor activity in osteosarcoma treatment (Yang et al., 2020). Similarly, PEG/PCL block polymers can be used to prepare injectable thermo-responsive hydrogels, which can aid drug delivery and effectively alleviate the side effects of chemotherapeutic drugs in normal tissues (Deng et al., 2019). Zhang et al. prepared PEG/poly((L)-leucine thermosensitive hydrogels for loading regorafenib and BMS202 (Zhang et al., 2021). In the CT26 *in situ* CRC tumor mouse model, the dual-drug hydrogel significantly alleviated the inhibitory TME and demonstrated promising therapeutic effects. The characteristics of the composite hydrogel, such as the ease of regulating its solubility, drug loading, degradation rate, and other properties, make it promising as an excellent DDS for tumor treatment.

The application of most synthetic polymers in antitumor drug delivery has challenges. For example, the drug release rate of synthetic polymers as DDS is affected by the polymer structure, chemical properties, and other factors, making it challenging to precisely control the rate and mode of drug release. In addition, some synthetic polymers may affect the stability of the drug, such as by degrading or inactivating the drug. This may interfere with the activity and efficacy of the drug in the body. Composites that combine the advantages of both natural and synthetic polymers have garnered the interest of researchers. Yuan et al. prepared a self-repairing hydrogel using PEG and chitosan that exhibited excellent loading efficiency for gemcitabine and DOX (Huang et al., 2022).

### 4.3. Green synthesis method for preparing hydrogel materials

In addition to utilizing natural materials such as chitosan, sodium alginate, and cellulose, crosslinking methods, solvents, and sustainable preparation processes are also very important for preparing green drug-loaded hydrogels. To better meet the needs of clinical applications, the preparation methods of drug-loaded hydrogels are constantly evolving and innovating. The green preparation methods of hydrogels, such as adopting technologies and processes that are environmentally friendly, sustainable, and have less impact on the environment and human health, have increasingly attracted the attention of researchers (Ho et al., 2022). These methods usually focus on reducing the use of harmful chemicals, lowering energy consumption, and improving the sustainability of the production process.

Ionic crosslinking technology constructs hydrogel networks through natural interactions between ions without the use of harmful chemical crosslinking agents, thereby reducing the potential risks to the human body and the natural environment (Mueller et al., 2022). Moreover, this reaction proceeds in a relatively mild environment, which helps maintain the stability of drugs. Therefore, ionic crosslinking technology meets the growing demand for environmentally friendly and biocompatible materials in the current medical field. For example, the hydrogel prepared by crosslinking sodium alginate and calcium ions is a typical example of green crosslinking. Shen et al. developed a sodium alginate hydrogel composed of elesclomol-Cu and galactose. This hydrogel can be crosslinked with physiological calcium ions (Ca^2+^) to form a hydrogel and control the release of elesclomol-Cu^2+^ and galactose to induce persistent cuproptosis (Shen et al., 2023). It is used for radio-immunotherapy of CRC and further prolongs the survival of tumor-bearing mice in local and metastatic tumors.

Similarly, the formation of hydrogel networks induced by physical methods such as temperature, light irradiation, or pressure can also avoid the introduction of toxic chemical crosslinking agents. Thermosensitive hydrogels can exhibit reversible solution-gel transition within a specific temperature range, and hydrogels undergo solubilization to attain a gel state when the ambient temperature exceeds the lower critical solution temperature. The PLEL hydrogel has excellent injectability and thermosensitivity. Wang et al. prepared an OxP/R848@PLEL hydrogel drug delivery system, which can effectively treat diffuse peritoneal metastasis in CRC patients through intraperitoneal injection (Wang et al., 2023). Light irradiation is another attractive method for inducing hydrogel crosslinking. As an external stimulus, light can induce structural changes in hydrogel networks carrying photosensitizers, thereby leading to drug release (Yew et al., 2024).

In addition, enzyme-catalyzed crosslinking is one of the commonly used green methods for synthesizing hydrogels. The crosslinking of hydrogels is achieved through enzyme-catalyzed reactions, which have advantages such as high efficiency, strong specificity, and mild reaction conditions (Li et al., 2020). At the same time, click chemical reactions can rapidly and efficiently synthesize hydrogels in an aqueous phase. Such reactions usually have high selectivity and low toxicity (Li & Xiong, 2022). Supramolecular hydrogels are constructed based on supramolecular chemistry principles using supramolecular compounds such as cyclodextrin and crown ether. These compounds have good biodegradability and biocompatibility (Gao et al., 2024).

The green synthesis methods of these hydrogels show advantages in biocompatibility, controllability, and potential for targeted applications, making them more attractive in a wide range of medical research and clinical applications.

## 5. Application of carrier-based hydrogels in CRC treatment

As an emerging drug carrier with superior biocompatibility, hydrogels can achieve controlled and targeted drug release, reduce drug-induced adverse effects, and enhance the therapeutic efficacy of drugs. These properties make hydrogels effective treatment agents for CRC.

### 5.1. Hydrogel-Mediated chemotherapy

Chemotherapy is known as an important treatment for CRC. As a systemic treatment, anti-cancer drugs used in chemotherapy can eliminate cancer cells or limit their growth and spread. In patients with CRC, chemotherapy is commonly administered before or after surgery to reduce the tumor size, improve the success rate of surgical resection, prevent recurrence and metastasis, and provide relief for patients with advanced disease stages (Woo & Jung, 2017). However, chemotherapeutic drugs exert toxic effects on normal cells while eliminating tumor cells, which leads to a series of adverse reactions, such as nausea, vomiting, hair loss, and fatigue (Chen et al., 2018). Hydrogels, as carriers of chemotherapeutic drugs, are promising agents for increasing the delivery efficiency and reducing the toxicity of drugs through their high drug-loading capacity, controlled release, and targeted effects.

**Figure 1.**
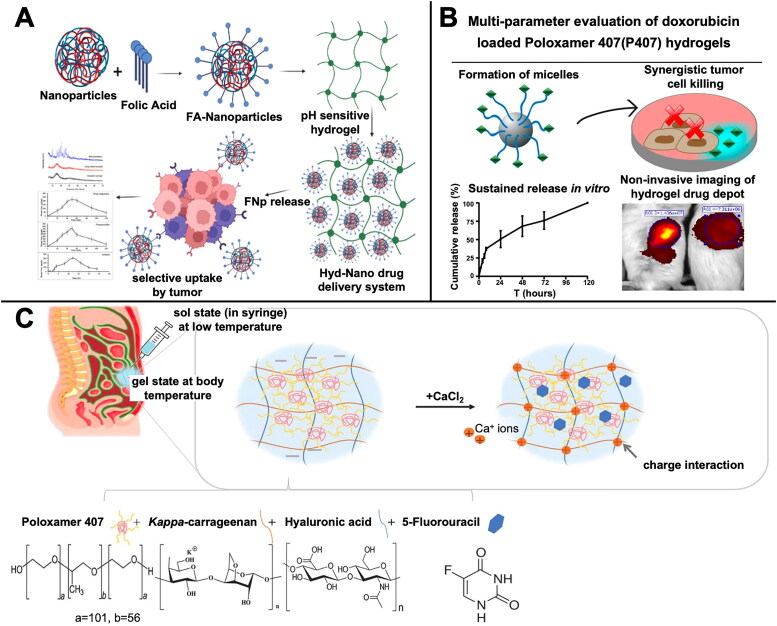
Hydrogel-mediated chemotherapy. A) Oral delivery of chemotherapeutic drugs is aided with pH responsive hydrogels loaded with folic acid modified nanoparticles and the hydrophobic drug diferuloylmethane. Reprinted with permission from ref (Abbasi et al., 2023). ©by 2023 Elsevier; B) P407 hydrogel loaded with DOX can significantly enhance its anti-tumor effects and be observed by noninvasive imaging. Reprinted with permission from ref (Chung et al., 2020). ©by *chih kit Chung* et al.; C) HA/kCGN/P407 cross-linked hydrogel can be applied for intraperitoneal delivery of 5-Fu for the treatment of peritoneal metastases of CRC and exerts anti-peritoneal adhesion. Reprinted with permission from ref (Dinh et al. 2022). ©by 2023 Elsevier.

Chemotherapeutic agents are commonly administered to patients either intravenously or orally. A key limitation of using the intravenous route is that it may lead to complications, such as uncertainties in the drug concentration in the blood, nonspecific delivery of the drug, and poor patient compliance (Wang et al., 2023). The oral route is considered the most favorable alternative to the intravenous route in such cases. The major challenge in the use of hydrogel drug carriers for oral administration is the maintenance of stable drug release and stable absorption in the GIT. Hydrogel drug carriers with pH sensitivity are an excellent solution to this problem. Hydrogels with weakly acidic groups remain tightly packed in the acidic environment of the stomach and GIT without releasing the drug and specifically target the colon cells to release the drug, as the pH increases in the lower part of the GIT. Ullah et al. used free radical polymerization to crosslink the pH-sensitive acrylic acid derivative 2-acrylamido-2-methylpropane sulfonic acid with the natural polymer gelatin to prepare pH-responsive gelatin-based hydrogels loaded with oxaliplatin, a first-line chemotherapeutic drug for CRC (Ullah et al., 2019). The hydrogels showed alterations in swelling patterns and drug release in response to pH changes in the surrounding environment and were found to exert dose-dependent cytotoxic effects on Vero, MCF-7, and HCT-116 cells. Oral tolerance studies conducted on rabbits indicated no significant signs of oral toxicity. Besides the use of pH-sensitive hydrogels to aid targeted drug release in the colon, several active modifications based on receptor and ligand binding have been used in investigations on hydrogels in oral drug therapy for CRC. Folic acid is considered a cancer-targeting ligand that binds to folate receptors overexpressed in the epithelium of several tumor cells, including CRC, for targeted drug delivery (Purohit et al., 2019). Abbasi et al. prepared a nanoparticle-loaded pH-responsive hydrogel system in which FA-modified sodium alginate nanoparticles loaded with the hydrophobic drug diferourylmethane were incorporated into a pH-responsive hydrogel (Figure 1A) (Abbasi et al., 2023). The DDS maintained an effective concentration of diferourylmethane over an extended period. Greater drug uptake was also observed in folate receptor-positive HeLa cells.

The injectable hydrogel, besides being administered via the oral route, can be administered directly into the periphery of the tumor, where it maintains a high drug concentration in the tumor tissue for a long period, effectively reducing the associated adverse effects. Chemotherapeutic drugs injected directly into the tumor are eliminated rapidly and cannot facilitate effective tumor treatment. As an excellent drug carrier, hydrogels can maintain the long-term stable release of drugs. Thus, they can help avoid the inconvenience of administering multiple injections. To load the antitumor drug DOX, Chung et al. prepared biodegradable injectable in situ hydrogels using Poloxamer 407 (P407), a polymer composed of a poly(ethylene oxide)-poly(propylene oxide)-poly(ethylene oxide) triblock copolymer with excellent temperature-sensitive properties. The P07 hydrogel facilitated the release of DOX for more than 120 hours, promoted the sustained action of the drug, and helped reduce the frequency of administration (Figure 1B) (Chung et al., 2020). Injectable *in situ*-forming hydrogels can ensure the sustained release of anticancer drugs at the tumor site without causing excess systemic side effects. Fiorica et al. prepared DOX-loaded *in situ*-forming hydrogels based on HA derivatives for sustained drug release in a 3D tumor cell culture model using β-cyclodextrin as a cross-linking agent. Findings from *in vivo* experiments also indicated their ability to inhibit tumor proliferation without inducing unnecessary drug accumulation in organs, such as in the heart (Fiorica et al., 2020).

Peritoneal adhesions and peritoneal metastases following CRC surgery are also clinical challenges that should be addressed urgently. The combined administration of anti-adhesion agents and anticancer drugs can help achieve local chemotherapeutic effects and may be a preferred option for systemic chemotherapy combined with intraperitoneal anti-adhesion therapy. LL37 is an endogenous tumor suppressor peptide, and its expression is significantly suppressed in human CRC tissues. Fan et al. used thermosensitive polylactic acid hydrogels loaded with paclitaxel and LL37 peptide nanoparticles, which were experimentally demonstrated to significantly inhibit the growth of HCT116 peritoneal carcinomas in loaded mice and effectively prolong survival (Fan et al., 2015). Dinh et al. prepared thermosensitive physically cross-linked hydrogels using P407, HA, and kappa-carrageenan (kCGN), in which HA and kCGN were crosslinked with Ca^2+^. The hydrogel had anti-adhesive properties and inhibited inflammation (Figure 1C) (Dinh et al., 2022). The drug carrier could promote the *in situ* release of the loaded 5-Fu to exert additional local antitumor effects while it was injected into the tumor site as an anti-adhesion agent. Hydrogels loaded with multiple drugs have also garnered the attention of researchers in the treatment of CRC peritoneal metastases. Luo et al. prepared HA hydrogels loaded with 5-Fu, cisplatin, and paclitaxel that exhibited strong therapeutic effects, such as ascites reduction and tumor growth and metastasis inhibition in a mouse model of CRC peritoneal metastasis (Luo et al., 2020). The use of agents such as mitomycin C and curcumin in hydrogel loading-related CRC treatment studies has also been emphasized (Stagnoli et al., 2023; Wintjens et al., 2023).

### 5.2. Hydrogel-Mediated radiation therapy

Radiotherapy is one of the most important aspects of comprehensive CRC treatment. The normal mucosa of the intestinal tract is sensitive to radiation and susceptible to damage. Biocompatible hydrogels can be used as protective spacers for the sex organs during radiotherapy. Achard et al. conducted a pilot trial in which a male and a female patient with rectal cancer were implanted with *TraceIT*^®^ hydrogel positioned between the rectum and the prostate/vagina. In the female patient, the hydrogel spacer effectively reduced the radiation dose to the vagina (Achard et al., 2021). Hydrogels capable of *in situ* formation can adhere to the surface of the target site and facilitate drug release and local action. Moussa et al. used hydroxypropylmethylcellulose hydrogels loaded with adipose-derived mesenchymal stromal cells (Ad-MSCs) that could be formed *in situ* and injected into the lesion periphery by colonoscopy. In a rat model of radiation-induced severe colonic injury, Ad-MSCs could secrete trophic factors, effectively improving the damaged colonic epithelial tissue and reducing local macrophage infiltration (Moussa et al., 2017).

**Figure 2.**
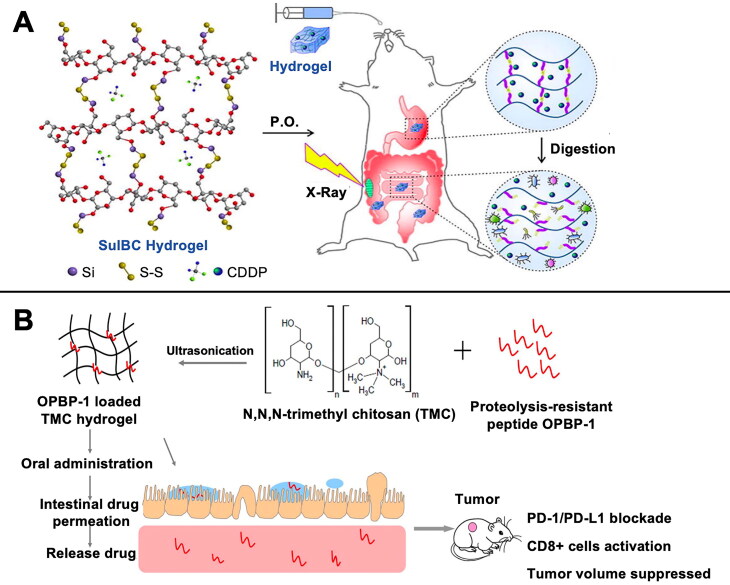
Hydrogel-mediated radiotherapy and immunotherapy. A) CDDP@SulBC hydrogel could accelerate the release of CDDP by degradation in reducing TME, increase X-ray-induced double-strand breaks, trigger G2/M-phase arrest, and enhance radiosensitivity to CRC cells. Reprinted with permission from ref (Shi et al., 2022). ©by 2022 Elsevier B.V. B) Trimethyl chitosan hydrogel-loaded oral PD-1/PD-L1 blocking peptide OPBP-1 was performed for CRC immunotherapy. Reprinted with permission from ref (Li et al., 2021). ©by 2021 Elsevier B.V.

Methods to enhance the radiotherapeutic sensitivity of CRC cells while protecting normal intestinal tissues constitute an integral part of our research. Radiotherapy sensitivity refers to the degree of response of tumor cells to radiation therapy, and the level of sensitivity directly determines the effects and prognosis of radiotherapy. Tumor cells in different cell cycle stages exhibit distinct sensitivities to radiation. Tumor cells in the S and M phases are more sensitive to radiotherapy, whereas those in the G0 and G1 phases are relatively less sensitive (Ben Barouch et al., 2020). Based on this, Shi et al. prepared sulfhydryl cross-linked bacterial cellulose hydrogels (CDDP@SulBC hydrogels) loaded with the chemotherapeutic drug cisplatin and used them in the precision radiotherapy of CRC (Figure 2A) (Shi et al., 2022). Bacterial cellulose has a large surface area, superior mechanical strength, and favorable biocompatibility. Bacterial cellulose hydrogel, as an excellent drug carrier for oral administration, can help regulate the metabolism of the intestinal flora, reduce intestinal inflammation, and maintain the intestinal mucosal barrier function (Jordan et al., 2018; Patnode et al., 2019). Meanwhile, the sulfhydryl group at the end of the hydrogel imparts reduction responsiveness and promotes rapid drug release in a reducing tumor environment. CDDP@SulBC hydrogel applied to tumor cells can induce G2/M phase arrest and increase X-ray-induced double-stranded breaks and radiosensitivity in CRC cells.

### 5.3. Hydrogel-Mediated immunotherapy

Immunotherapy is an emerging trend in tumor treatment research. It is based on the principle of stimulating immune responses against tumors. This is done by enhancing the immunogenicity of tumor cells and strengthening the ability of immune cells to eliminate tumor cells in collaboration with the immune system. The aim is to eliminate tumors and suppress tumor growth. Currently, immunotherapies that have received extensive attention include ICI, sequential immune cell therapy, cytokine therapy, and tumor vaccines (Aparicio et al., 2021; Ying Li et al., 2024). Bevacizumab, which targets VEGF, is approved for CRC treatment as it suppresses angiogenesis at the tumor site, depriving tumor tissues of oxygen and nutrients, and inhibits tumor progression. However, the the administration of bevacizumab intravenous injections bi-weekly over 30–90 min can be extremely inconvenient for patients (Xie et al., 2020). Lee et al. used an ABA triblock copolymer hydrogel of vitamin D-functionalized polycarbonate and polyethylene glycol as a sustained release bevacizumab drug carrier (Lee et al., 2015). In an HCT116 subcutaneous tumor model mice, the subcutaneous injection of bevacizumab-loaded hydrogel enhanced antitumor effects compared to those induced by the intravenous injection of bevacizumab, with significant drug accumulation at the tumor site. In a mouse model of peritoneal metastasis, the anti-metastatic efficacy of a single injection of hydrogel-delivered bevacizumab was comparable to that of four weekly intravenous injections. Thus, subcutaneously injectable hydrogel carriers can help reduce the frequency of injections and increase the convenience of and adherence to treatment.

PD-1 can potentially help regulate and limit the attack by the immune system on healthy tissues and is used by tumor cells to evade the immune system. PD-1 inhibitors suppress the growth and spread of tumors by blocking the binding between PD-1 proteins and their ligands, which enables immune cells to identify and attack cancer cells with greater effectiveness (Han et al., 2020). Monoclonal antibody peptides for blocking PD-1/PD-L1 exhibit excellent specificity and low immunogenicity. However, peptide drugs have an inferior half-life and face significant challenges in oral drug delivery, which considerably limits the effectiveness of their clinical application. Hydrogels can prolong the half-life of drugs and improve their bioavailability. Li et al. demonstrated the significant inhibition of CRC tumor growth and enhanced CD8^+^ T-cell infiltration and function in CT26 mice treated with N,N,N-trimethylchitosan hydrogels loaded with orally administered PD-L1-binding peptide 1 with proteolytic hydrolysis resistance (Figure 2B) (Li et al., 2021). Compared to intravenous administration, oral administration is more likely to be accepted by patients and is a major direction for future drug therapy research.

Natural killer (NK) cells may induce tumor cell lysis and apoptosis by releasing cytotoxins and perforins (Li et al., 2021). Some studies have shown that polysaccharides isolated from natural plants, such as mushrooms, exhibit excellent antitumor and immunomodulatory properties and can enhance the activity of NK cells, macrophages, and lymphocytes (Zong et al., 2012; El-Deeb et al., 2019). El-Deeb et al. used pH-responsive alginate/kappa carrageenan hydrogels loaded with novel mushroom polysaccharides. These activated NK cells demonstrated enhanced cytotoxicity against human colon cancer Caco-2 cells. These hydrogels can be used as an orally targeted DDS in CRC immunotherapy (El-Deeb et al., 2022). Tumor vaccines can induce immunity against specific tumors, and the cells commonly used as loaded tumor antigens are dendritic cells (DCs). Yang et al. prepared alginate hydrogels loaded with tumor cell lysates and the anti-angiogenic peptide Endostar, which significantly inhibited the tumor growth of MC-38 ruffed-tumor mice and increased the proportion of CD8^+^ T cells (Yang et al., 2022). Yu et al. prepared hydrogels dynamically cross-linked with dobby PEG and oxidized dextran using proteins extracted from tumors as antigens. The subcutaneous injection of these hydrogels into MC38 mouse tumor models triggered tumor-specific immune responses by recruiting DCs (Yu et al., 2021). Hydrogel-loaded vaccine immune adjuvants also showed strong effects in CRC treatment (Nkanga & Steinmetz, 2022).

### 5.4. Hydrogel-mediated hyperthermia, photothermal effects, and PDT

Tumor cells have limited tolerance to high temperatures. Therefore, increasing the temperature of local tumor tissues or the body in general using different means can help effectively eliminate tumor cells. This is considered a form of green therapy and used for treating several types of cancers, including CRC. Tumors have an imperfect tissue structure and poor heat dissipation ability and show a rapid rise in temperature. Rapidly growing tumor cells are sensitive to high temperatures. Thus, tumor cells are eliminated or gradually become apoptotic in response to thermotherapy, whereas normal tissues are not damaged. Research indicates that hyperthermia can directly eliminate tumor cells by inhibiting DNA, RNA, and protein biosynthesis, damaging cell membrane integrity, and suppressing tumor respiration. Concurrently, hyperthermia can also enhance the effects of radiotherapy and chemotherapy and physiological immune activity and inhibit tumor progression. Various modalities can be used to increase the temperature of the body or tumor tissues, such as radiofrequency, microwave, ultrasound, and laser, among others. Through these modalities, high temperature and its secondary effects can be used to treat tumors. Radiofrequency ablation can generate heat through high-frequency electrical currents and is commonly used to treat liver and lung metastases of CRC (Karaoğlan et al., 2024). High-intensity focused ultrasound (HIFU) is used for the minimally invasive treatment of tumor tissues. In HIFU, high-intensity ultrasound waves are used to kill tumor cells in the target area without damaging adjacent healthy tissues. HIFU can also facilitate the release of liposome-encapsulated drugs. The network structure and high water content in hydrogels can contribute to the rapid and uniform transfer of heat while protecting the adjacent healthy tissues. These hydrogels can be used as drug carriers loaded with thermal sensitizers for tumor therapy. Jeong et al. prepared PEG liposomes loaded with indocyanine green and DOX and used HIFU to facilitate the targeted release of the drugs. This treatment demonstrated efficient therapeutic effects in a CT26 mouse tumor model (Jeong et al., 2016). Magnetic hyperthermia therapy (MHT) is also an effective noninvasive method to treat tumors and is not restricted by the depth of penetration (Yan et al., 2021). Chen et al. prepared an injectable magnetic hydrogel loaded with a potent iron death inducer, RSL3. Enhanced iron-dependent cell death could reduce the MHT-triggered tumor thermoresistance effect by impairing heat shock protein 70, which is a synergistic strategy for the effective treatment of CT-26 tumors in mice (Chen et al., 2023).

**Figure 3.**
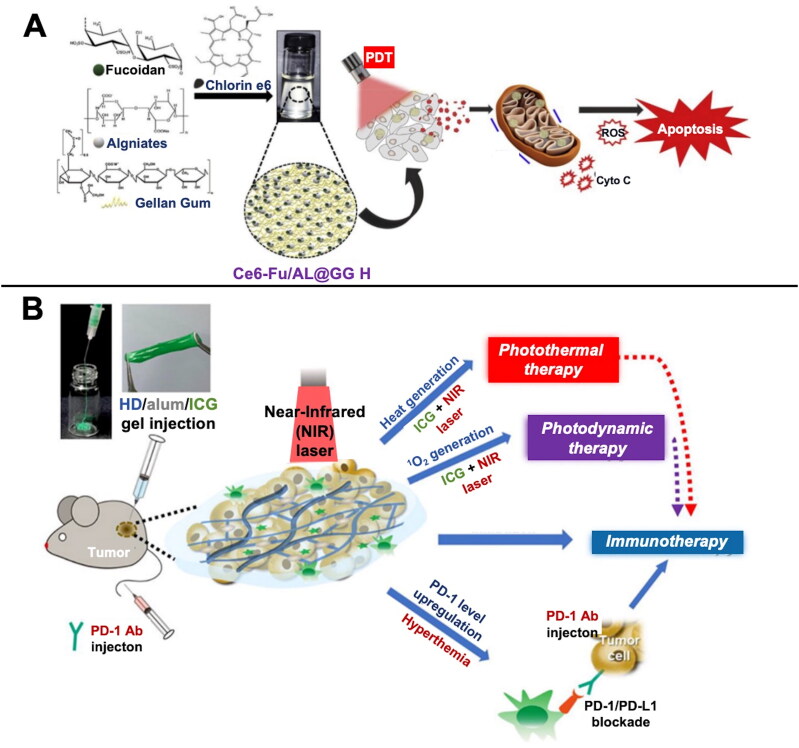
Hydrogel-mediated PDT and combined therapy. A) Ce6-Fu/AL@GG-based hydrogel can mediate mitochondrial ROS production under NIR irradiation, leading to HT-29 cell apoptosis. Reprinted with permission from ref (Karuppusamy et al. 2019). ©by 2019 Elsevier; B) HD/alum/ICG hydrogel loaded with PD-1 antibody can be administered under NIR irradiation to treat tumors by a combination of PTT, PDT and immunotherapy. Reprinted with permission from ref (Kim et al. 2023). ©by 2023 Elsevier B.V.

PTT is a highly regarded tumor treatment method based on hyperthermia. In PTT, special photosensitizers (PS) injected at the tumor site generate heat under a specific wavelength laser beam to damage the surrounding tissues for treating the tumor. PS commonly prescribed for tumor treatment using PTT include chlorin e6 (Ce6), titanium dioxide nanoparticles, and gold nanoparticles. Karuppusamy et al. prepared a fucoidan/arginine-based hydrogel with Ce6 as the PS, which could significantly inhibit the growth of HT-29 cells under laser irradiation by inducing cellular reactive oxygen species (ROS) production and mitochondrial damage to kill tumor cells (Figure 3A) (Karuppusamy et al., 2019). Indocyanine green as a PS has also been used in studies on the use of PTT for CRC treatment (Qing et al., 2021). The high temperature used in PTT may cause unnecessary damage to adjacent healthy tissues, whereas the low-temperature high temperature (<45 °C) of NIR laser irradiation can destroy tumor cells at a relatively lower temperature with considerable safety (Yoo et al., 2013).

PDT, as an emerging minimally invasive therapeutic approach, is a promising method for improving the outcomes of CRC treatment. PDT treatment involves the selective accumulation of PS in cancer cells and the subsequent exposure to specific wavelengths of light to activate the PS, which induces the synthesis of ROS that destroy local tumor cells (Agostinis et al., 2011). Hydrogels can facilitate the delivery of PS to the tumor site, generating ROS to kill tumor cells under stimulation by external light. Ouyang et al. constructed multifunctional *in situ* hydrogels using sodium alginate and Ca^2+^, loaded with inks that act as PS and an azo initiator of 2,2′-azobis[2-(2-imidazolin-2-yl)propane] dihydrochloride (AIPH) that can induce ROS synthesis for tumor treatment (Ouyang et al., 2019). NIR-II, which has greater penetrative potential, was used as an exogenous light stimulus in the experiment to promote local heat generation from the ink. This triggered the decomposition of AIPH and the synthesis of ROS in large quantities, which synergistically killed the tumor cells. Concurrently, the ink also produced strong photoacoustic signals that could be used for surveillance.

### 5.5. Hydrogel-mediated combination therapy

Single chemotherapeutic agents can temporarily alleviate cancer progression but are prone to tumor resistance. This has led to research on tumor treatment using a combination of chemotherapeutic agents to overcome drug resistance. The key focus in this area of biomedicine is achieving long-lasting efficacy by combining multiple chemotherapeutic agents. Gong et al. used a thermosensitive PEG/PCL hydrogel for the simultaneous delivery of 5-Fu and paclitaxel. Its intraperitoneal injection effectively inhibited the growth and metastasis of CT26 peritoneal carcinoma in mice, exhibiting a long-lasting drug release potential (Gong et al., 2012). Drugs that can enhance the effects of chemotherapeutic agents or mitigate their adverse effects can also be used in combination with chemotherapeutic agents. Ren et al. used thermosensitive hydrogels loaded with oxaliplatin and tannic acid, which was believed to alleviate the side effects of chemotherapeutic drugs, to treat the peritoneal metastasis of CRC; the hydrogens were injected intraperitoneally. The treatment effectively improved the quality of survival and increased the survival of model mice (Ren et al., 2019). Chitosan hydrogels loaded with low-bioavailability curcumin and silver nanoparticles effectively inhibited the growth of Caco-2 human CRC cells induced by visible light and may be used as diagnostic probes (de Freitas et al., 2020). Keshavarz et al. prepared alginate hydrogels co-loaded with cisplatin and gold nanoparticles. While effectively providing sustained-release drug therapy, CT imaging technology can be used to monitor the distribution and metabolism of hydrogels in vivo in real time (Keshavarz et al., 2018).

The combination of PTT and chemotherapeutic drugs exhibits a superior collective antitumor effect, and the thermal transformative effect of PTT can promote protein denaturation in tumor cells, inhibit the activity of DNA repair enzymes in tumor cells after chemotherapy, induce the death of cancer cells, and enhance the therapeutic efficacy of chemotherapeutic drugs. Concurrently, localized high heat can effectively improve the permeability of the cell membrane, accelerate the uptake of drugs by tumor cells, and improve the solubility of chemotherapeutic drugs in the TME and the release of drugs. Wang et al. prepared an *in situ* injectable hydrogel containing gold nanorods and paclitaxel that effectively combined the advantages of chemotherapy and PTT therapy. This hydrogel showed excellent treatment effects in multidrug-resistant CRC, exhibiting prolonged palliation while attenuating the toxic effects of the drug. It showed promise as a single-administration, long-term tumor control agent (Wang et al., 2018). The high levels of heat generated by PT can also effectively enhance immune function. Based on this phenomenon, Kim et al. prepared HA/dopamine hydrogels for immunophototherapy (Figure 3B) (Kim et al., 2023). They loaded HA/dopamine hydrogels with both indocyanine green and an anti-PD-1 antibody, which could induce local hyperthermia to generate ROS under NIR irradiation and enhance the tumor cell-eliminating ability of CD8^+^ T cells present on tumor cells to inhibit the progression of primary tumors and distantly metastatic tumors. Liang et al. developed a sequential DDS based on a thermosensitive chitosan/oxidized dextran hydrogel that promoted the local release of 5-Fu and methotrexate through hyperthermia generated by the photothermite MoS_2_ under NIR irradiation for the synergistic treatment of CRC using chemotherapy and PTT (Liang et al., 2023).

Radiation therapy may also promote the release of tumor-specific antigens for recognition by immune cells while killing local tumor lesions. This enhances the efficacy of immunotherapy. Shen et al. prepared a sodium alginate hydrogel composed of Cu^2+^ and galactose, which could cross-link with Ca^2+^ at local tumor lesions, inducing the cuproptosis of CRC cells and reducing PD-L1 expression on the surface of the tumor cells. This effectively improved the sensitivity of the tumor to radiotherapy and immunotherapy (Shen et al., 2023). The combination between different immunotherapy modalities is also essential in CRC treatment. Molecular targeted therapy is among the most important therapeutic modalities for CRC. However, its therapeutic efficacy is limited by the upregulation of PD-L1 on the surface of tumor cells. To study the effects of combined molecular targeted therapy and ICI, Zhang et al. prepared PEG/poly((L)-leucine thermosensitive hydrogels for loading regorafenib and BMS202. In the CT26 *in situ* CRC tumor mouse model, the dual-drug hydrogel significantly alleviated the inhibitory effects of the TME and demonstrated promising therapeutic effects (Zhang et al., 2021).

### 5.6. Serving as culture medium for CRC research

In addition to serving as a drug carrier to assist in the treatment of CRC, hydrogels, as a material with unique properties, have shown great potential in *in vitro* 3D culture and provided a powerful tool for CRC research (Cadamuro et al., 2023). The extracellular matrix (ECM) in the tumor microenvironment in vivo is composed of multiple components, including collagen, fibronectin, HA, etc (Goenka et al., 2023). Hydrogels can simulate the structure and function of ECM by incorporating these components or their analogs. For example, hydrogels containing collagen can provide sites for cell adhesion and promote cell growth and differentiation. At the same time, components such as hyaluronic acid can regulate the porosity and permeability of hydrogels and affect the diffusion of nutrients and metabolites. In addition, the interaction between cells in the tumor microenvironment (TME) also plays a key role in tumor development and treatment response (Roy et al., 2023). Hydrogels can support the co-culture of multiple cell types, including tumor cells, immune cells, fibroblasts, etc., thereby simulating the interaction between cells *in vivo.* Luo et al. encapsulated patient-derived organoid (PDO) CRC models in HA/gelatin hydrogels and co-cultured them with cancer-associated fibroblasts (CAF) derived from patient tumor tissues. Experiments have proved that this 3D culture model can help evaluate standard therapeutic drugs and promote the realization of personalized cancer medicine (Luo et al., 2021).

### 5.7. Current clinical development of hydrogels for CRC treatment

As a drug carrier with great potential, some drug-loaded hydrogels have entered the clinical trial stage of CRC treatment. We searched for clinical trial information registered in Clinical Trials (https://clinicaltrials.gov/) and the International Clinical Trials Registry Platform (ICTRP Search Portal (who.int)) and found that a total of four drug-loaded hydrogels have entered the clinical trial stage and are recruiting patients. Relevant information is summarized in Table 1. As a new drug delivery system, drug-loaded hydrogels have shown great potential in the treatment of CRC. With the continuous deepening of research, the performance of drug-loaded hydrogels will be continuously optimized, and its application prospects in CRC treatment will be broader.

**Table 1.** Clinical trials related to hydrogels are designed to aid in the treatment of CRC.

ID	Responsible party	Official title	Intervention / treatment	Brief	Primary outcome measures	Phase	Time
NCT06385418	The Second Hospital of Nanjing Medical University	Fluorouracil Treatment Via Colon for Colorectal Cancer: an Exploratory Study	Drug: Colonic local administration of fluorouracil with enhanced adhesion	Utilize the colonoscopic enteral tube to flexibly cover the entire colon and combine it with a thermosensitive gel to enhance the adhesion of 5-Fu.	Objective response rate	2	2024/5/16-Up to now
NCT03258541	University Hospital, Geneva	TraceIT^®^ Hydrogel Spacer Injections for Vagina and Erectile Bundles Sparing in Rectal Cancer Patients Treated With Neoadjuvant Radiotherapy: a Feasibility Study	Device: TraceIT^®^； Radiation: Volumetric Arc Therapy (VMAT)； Procedure: Surgery	Study the feasibility and effectiveness of the injectable hydrogel spacer TraceIT^®^ in preserving the vagina/prostate in patients with rectal cancer during radiotherapy.	Clinical performance	N/A	2017/1/15-2020/05
NCT04595266	Grupo Espanol Multidisciplinario del Cancer Digestivo	Chemoembolization (Lifepearls-Irinotecan) in Patients With Colorectal Cancer and Metastatic Disease (LIVERPEARL)	Drug: FOLFOX regimen Biological: Anti-EGFR or Bevacizumab Drug: LIVERPEARLS-Irinotecan	Utilize LIFEPEARLS^®^ to form hydrogel microspheres of complex irinotecan, helping irinotecan penetrate deeper into tumor tissues for the treatment of CRC and mCRC.	Objective response rate	2	2021/6/29-Up to now
NCT04062721	Hôpitaux de Paris	Local Immunomodulation After Radiofrequency of Unresectable Colorectal Liver Metastases (LICoRN-01)	Drug: Chemotherapy Procedure: Radiofrequency ablation (RFA) Drug: In situ immunotherapy	Study the feasibility and tolerance of using thermosensitive hydrogel combined with two immunomodulators, GMCSF and Mifamurtide, for unresectable liver metastases of colorectal cancer.	Incidence of treatment-emergent adverse events	1	2021/6/1-Up to now

## 6. Stimuli-responsive hydrogels and application mechanisms

Hydrogels have been extensively used as DDS in cancer therapy research. Smart hydrogels that respond to temperature, light, pH, redox environments, and magnetic properties can also respond to changes in the TME or the surrounding environment to facilitate targeted drug release and control. Smart-responsive hydrogel drug-controlled release systems commonly incorporate *in situ* cross-linking polymerization-embedding technology or physical adsorption technology to load drugs. Once the hydrogel is injected into the organism and senses changes in external stimuli (such as pH, temperature, light, electricity, and magnetism, among others) and environmental factors in the lesion, it responds by altering its structure or properties, including its hydrophilic and hydrophobic properties. This leads to the dissolution or contraction of the hydrogel and the subsequent release of the drug and helps achieve targeted, regular, and quantitative drug release (Table 2).

**Table 2.** Stimuli-responsive hydrogels for CRC drug delivery.

Hydrogel	Responsive	Drug	Delivery Route	Research Models	Highlight	Ref
OXA/TA NPs-H	Thermal	OXA; TA	Intraperitoneal injection	CT26 peritoneal carcinomatosis mice model	The two-drug combination limited the growth of peritoneal carcinomas and prolonged survival time in mice.	(Ren et al., 2019)
PEG-PLLeu	Thermal	Regorafenib; BMS202	In situ injection	orthotopic rectal cancer model (CT26-Luc)	The combination of targeted drugs and ICI produces a synergistic effect in the *in situ* treatment of rectal cancer without significant toxicity.	(Zhang et al., 2021)
PLA-L35-PLA	Thermal	Docetaxel	Intraperitoneal injection	HCT116 peritoneal carcinomatosis mice model	Significantly inhibited peritoneal cancer growth and prolonged survival in mice *in vivo.*	(Fan et al., 2015)
HA/(kCGN/P407	Thermal	5-Fu	Intraperitoneal injection	Sprague-Dawley rat model	Sustained drug release to enhance local anti-tumor capacity	(Dinh et al., 2022)
PTX-micelles-Fu-hydrogel	Thermal	PTX; Fu	Intraperitoneal injection	CT26 peritoneal carcinomatosis mice model	Significantly increases the concentration and residence time of PTX and Fu in the peritoneal fluid.	(Gong et al., 2012)
PNiPenPH	Thermal	DOX		HCT116	Long-lasting DOX administration	(Carreño et al., 2021)
P407/P188/alginate/5-Fu	Thermal	5-Fu	Intratumoral injection; Subcutaneous application	Ectopic CT26 murine models	Enhanced local administration of 5-Fu to significantly slow tumor growth	(Al Sabbagh et al., 2020)
OxP/R848@PLEL	Thermal	OXA	Intraperitoneal injection	CT26 peritoneal carcinomatosis mice model	The powerful combination of chemotherapy and immunotherapy effectively eradicated peritoneal metastases, completely inhibited the production of ascites.	(Wang et al., 2023)
CS/β-GP	Thermal	Cisplatin	Intraperitoneal injection	HCT116; Rats	Enhanced cytotoxic effects and reduced toxicity to normal tissues	(Abdel-Bar et al., 2016)
HACPN-DOX	Thermal	DOX	Intraperitoneal injection	CT26; BALB/c mice (peritoneal carcinomatosis animal model)	Effective inhibition of peritoneal metastasis growth and prevention of postoperative peritoneal adhesions	(Chen et al., 2018)
PTX/PECT	Thermal	PTX	Intraperitoneal injection	CT26 peritoneal carcinomatosis mice model	Effectively improves the bioavailability of PTX.	(Xu et al., 2016)
OXA-MC	Thermal	OXA	Intraperitoneal injection	CT26 peritoneal carcinomatosis mice model	Excellent biocompatibility and thermal sensitivity, and effective inhibition of tumor growth.	(Yang et al., 2024)
mPEG-b-PELG	Thermal	Cisplatin; CA4P	Intratumoral injection	CT26 mice model	Effective anti-tumor by co-administration.	(Yu et al., 2019)
CS	Thermal	Cisplatin; 5-Fu	Intraperitoneal injection	CT26 peritoneal carcinomatosis mice model	Effectively inhibits tumor growth and metastasis, prolongs survival time and improves the effect of chemotherapy.	(Yun et al., 2017)
Cs/AAc/AMPS	pH	5-Fu	–	–	The pH-responsive hydrogel assists in reducing the release of the drug in the stomach pH.	(Ghobashy et al., 2021)
FNPs-hydrogels	pH	Diferourylmethane	Oral administration	Hela, A-549; Rabbits	Synergistic delivery of hydrophobic drugs to CRC cells via oral route through FA targeting and pH responsiveness.	(Abbasi et al., 2023)
CMC/PVP-g-poly(AA)	pH	Fu	Oral administration	Hela; Rabbits	Potential for safe and site-specific continuous Fu delivery.	(Hanan et al., 2024)
HP-β-CD-g-MAA	pH	Cytarabine	Oral administration	Rabbits	Excellent biocompatibility and effective in prolonging the plasma half-life of the drug.	(Batool et al., 2022)
NAR-Sp	pH	NAR		HCT116	Sustained delivery of NAR, a hydrophobic anticancer drug, and demonstration of superior antitumour effects.	(Md et al., 2022)
CMC/GQDs	pH	DOX	–	HT-29	Potential applications for fluorescence imaging in addition to pH-responsive DOX release.	(Rakhshaei et al., 2020)
HP-β-CD/agarose-g-poly(MAA)	pH	Capecitabine	Oral administration	Rabbits	Enhancement of the antitumour effect of capecitabine and effective reduction of its toxic side effects.	(Rehman et al., 2021)
CaCO_3_/MTX/Alg/CMC/Asp	pH	MTX; Asp	–	SW480	The dual drug delivery system protects MTX from gastric and small intestinal absorption, and Asp helps alleviate adverse patient reactions.	(Sheng et al., 2021)
PpIX/MnO_2_/GSH inhibitor	redox	SDT	Intratumoral injection	Subcutaneous CRC mouse models.	GSH inhibitors could block the synthesis of intracellular GSH, which further enhanced the action of SDT.	(Chen et al., 2022)
cRGD-9R-PA ss	redox	siRNA	–	CT26	Functional motifs such as the tumor-targeting peptide cRGD, and the redox-responsive disulfide bond were introduced into polyacrylamide nanohydrogels, and siRNAs were successfully delivered to tumor cells for silencing the target genes.	(Yu et al., 2024)
ALG/Fe_3_O_4_	light	PTT	–	CT26	Mediates the killing of tumor cells by PTT under near-infrared laser light.	(Ji & Wang, 2023)
GNRs-TPGS-PTX NC-gel	light	PTT; PTX; TPGS	Intratumoral injection	SW620; SW620 AD300 tumor-bearing mice	Combining PTT and chemotherapeutic agents for multidrug-resistant CRC results in long-term tumor control with a single dose.	(Wang et al., 2018)
ALG/AIPH	light	PDT; AIPH	Intratumoral injection	HCT116 subcutaneous tumor models	Killing of tumor cells by the synergistic action of low-temperature PTT and cytotoxic-free free radicals.	(Ouyang et al., 2019)
HD/alum/ICG	light	PDT; PD-L1 antibody	Intratumoral injection	CT26; CT26 mice model	Combining PTT and immune drugs for effective tumor treatment.	(Kim et al., 2023)
CHT/CS/CUR-AgNPs	light	CUR	–	Caco-2	Improved bioavailability of curcumin and potential as a fluorescent probe.	(de Freitas et al., 2020)
CS/PAA/Fe_3_O_4_	magnetic	5-Fu	–	simulation model	Enhanced drug stability for long-term controlled release in the colon and rectum.	(Amini-Fazl et al., 2019)
NPs/RSL3@AAGel	Magnetic	MHT	Intratumoral injection	CT26; CT26 mice model	Enhancement of MHT efficacy by iron death inducers.	(Chen et al., 2023)
PEC	Magnetic	Irinotecan	Intravenously injection	HCT116; HCT116 subcutaneous tumor models	Delivery of irinotecan is aided by the employment of PEC particles with superparamagnetic properties, which have promising tumor-targeting capabilities and improved anti-CRC effects.	(Wu et al., 2020)
MA-CMCS	Enzyme	Imatinib	Oral administration	LS174T; LS174T subcutaneous tumor models	Hydrolysis of MA-CMCS glycosidic bonds by enteric enzymes aids delivery of hydrophobic imatinib.	(Wang et al., 2022)
HEMA/MAA	Enzyme	5-Fu	–	HCT116	Hydrolysis of olsalazine in hydrogels using azoreductase in the gut promotes targeted release of 5-Fu.	(Ma et al., 2019)
CS-ID@NM	Ultrasound	SDT; ICIs	Oral administration	CT-26; orthotopic colon cancer mice model	Combination of targeted chemotherapy-tumor lysis-immunotherapy with CS hydrogels encapsulating ultrasound-responsive mesoporous manganese oxide and ICIs.	(Cao et al., 2022)
aCD47/aPDL1@PB-TA	ROS	LDFRT; ICB	Intraperitoneal injection	CT26 peritoneal carcinomatosis mice model	Hydrogels with peritoneal adhesion properties and ROS responsiveness were loaded with ICB drugs and promoted drug release induced by ROS generated by LDFRT.	(Liu et al., 2023)
CMCS-SA	pH/magnetic	DOX	–	HCT116	The pH responsiveness protects the drug from degradation in the gastric juice and accelerates drug release at the tumor site through the action of an external magnetic field.	(Fang et al., 2022)
Gel/(REG+NG/LY)	Thermal/ROS	REG; LY	Intratumoral injection	CT26; CT26 mice model	Thermal and ROS dual-responsive hydrogels enable improved adaptation to TME, enhance molecularly targeted therapeutic effects and amplify immune activation.	(Li et al., 2022)
BI-ES-FeAlg/DOX	Redox/ bacteria	DOX	Intravenously injection	CT26 BALB/c mice model	Being able to overcome physical barriers to assist drug targeting to accumulate in the tumor tissue.	(Zhang et al., 2022)
PDESSB30	pH/redox	siRNA	–	Caco-2	Dual-responsive hydrogels delivered functional siRNA to Caco-2 cells and achieved successful silencing of target genes.	(Liechty et al., 2019)

**Abbreviation:** CS: chitosan; β-GP: β-glycerophosphate; PNiPenPH: N-isopropylacrilamide and 4-penten-1-ol crosslinked with poly(ethylene glycol) diacrylate; DOX: doxorubicin; HACPN: poly(N-isopropylacrylamide); HA: hyaluronic acid; kCGN: kappa-carrageenan; P407: poloxamer 407; PTX: paclitaxel; OXA: oxaliplatin; TA: tannic acid; R848: resiquimod; PLEL: PDLLA-PEG-PDLLA; TRHS: thermoresponsive hydrogel systems; TPT: topotecan; SLNs: solid lipid nanoparticles; PECT: poly (ε-caprolactone-co-1,4,8-trioxa [4.6]spiro-9-undecanone)-poly(ethylene glycol)-poly (ε-caprolactone-co-1,4,8-trioxa [4.6]spiro-9-undecanone); MC: methyl cellulose; CA4P: combretastatin A4 disodium phosphate; PEG-PLLeu: poly (ethylene glycol)-block-poly ((L)-leucine); ICI: immune checkpoint inhibitor; FNPs: folic-acid conjugated nanoparticles; AAc/AMPs: (acrylic acid)-co-(2-acrylamido-2-methylpropane-sulfonic acid); CMC: carboxymethyl cellulose; PVP: polyvinylpyrrolidone; poly(AA): poly(acrylic acid); NAR: naringenin; GQDs: graphene quantum dots; MTX: methotrexate; Alg: alginate; MnO_2_: manganese oxide; PpIX: protoporphyrin IX; SDT: sonodynamic therapy; BI: *Bifidobacterium Infantis;* ES: endostatin; CS: chondroitin sulfate; AgNPs: silver nanoparticles; CUR: curcumin; ICG: indocyanine green; HD: hyaluronic acid-dopamine; AIPH: azo initiator of 2,2'-azobis[2-(2-imidazolin-2-yl)propane]dihydrochloride; GNR: gold nanorod; TPGS: D-alpha-tocopheryl PEG 1000 succinate; MHT: magnetic hyperthermia therapy; PEC: polyelectrolyte complexe; HEMA: hydroxyethyl methacrylate; MAA: methacrylic acid; REG: regorafenib; aCD47: anti-cluster of differentiation 47; aPDL1: anti-programmed cell death 1 ligand 1; TA: tannic acid; LDFRT: low-dose fractionated radiotherapy.

### 6.1. Thermo-responsive smart hydrogels

Heat serves as a stimulus that can be conveniently controlled both internally and externally in an organism. Thermosensitive hydrogels can exhibit reversible solution-gel transition within a specific temperature range, and hydrogels undergo solubilization to attain a gel state when the ambient temperature exceeds the lower critical solution temperature (LCST). Thermosensitive hydrogels can be used as a DDS in tumor therapy. The relatively high temperature of the tumor tissue can trigger the swelling of the hydrogel and promote the release of the drug at the tumor site. At the LCST between room and body temperatures, the hydrogel is capable of *in situ* gelation after injection and is highly suitable for application as a DDS in tumor therapy.

Both hydrophilic and hydrophobic groups are present in the thermosensitive hydrogel system. When the temperature is lower than the LCST, the hydrogen bonding between hydrophilic groups and water molecules in the hydrogel drug release system is dominant. A large number of water molecules enter the hydrogel drug release system, representing a solution state. With the increase in the ambient temperature, the thermosensitive group becomes insoluble in the dissolved state, and the hydrogen bonding between this group and water molecules weakens. The hydrogen bonding within the system molecules dominates, and the hydrophobicity of the polymer molecule chain within the system is strengthened. This destroys the solvent shell layer, and the drug loaded in the solvent shell layer dissolves with the water molecules and reaches the focal point of the lesion. Polymers such as poly(N-isopropylacrylamide), which are commonly available for the preparation of thermosensitive hydrogels, can accomplish sol-gel state transition at approximately 32 °C by introducing hydrophobic groups (Carreño et al., 2021). Al Sabbagh et al. used the reversible thermogel properties of polymerase P407 and P188 to load 5-Fu and added 1% w/v alginate to aid the sustained release of 5-Fu. They administered the agent *via* intratumoral and post-tumor resection subcutaneous injections to evaluate the therapeutic effects of this drug platform in a CT26 mouse model of CRC (Al Sabbagh et al., 2020).

Post-surgical peritoneal adhesions and advanced peritoneal metastases are major challenges in the clinical management of CRC. Thermo-responsive hydrogels show promising effects in the resoution of these issues. Cross-linking with HA and *kappa*-carrageenan in polyoxyethylene-based thermosensitive hydrogels used for delivering chemotherapeutic agents in CRC treatment can prevent post-surgical peritoneal adhesions (Dinh et al., 2022). Fan et al. used polylactic acid hydrogels loaded with docetaxel and LL37 peptides injected intraperitoneally for treating the peritoneal metastasis of CRC, which significantly inhibited the growth of peritoneal carcinomas and prolonged the survival of mice with HCT116 (Fan et al., 2015). Thermal-responsive hydrogels can also facilitate the effective simultaneous local co-delivery of multiple chemotherapeutic agents to increase efficacy and reduce toxicity. Ren et al. co-loaded tannic acid and oxaliplatin, which exert anticancer effects and mitigate the side effects of chemotherapeutic drugs, into a thermo-responsive PCL hydrogel. This hydrogel had an LCST and could undergo a solution-gelation transformation at body temperature after local injection (Ren et al., 2019).

**Figure 4.**
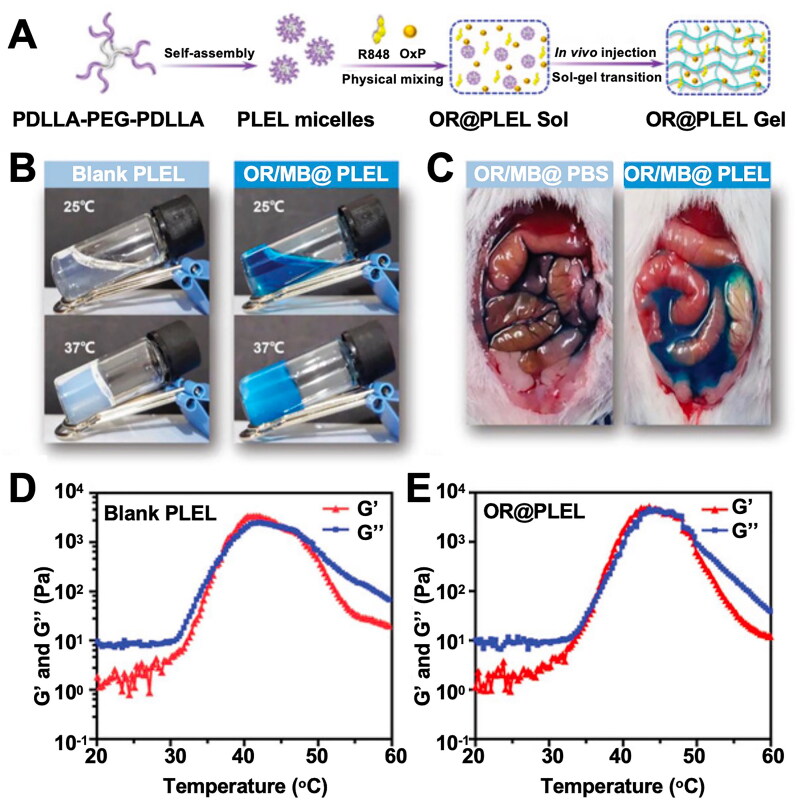
PLEL thermo-responsive hydrogel loaded with oxaliplatin and resiquimod for synergistic treatment of peritoneal metastases in advanced stages of CRC. A) Schematic of the preparation of or@PLEL; B) *In vitro* sol-gel transformations of blank gel and or/MB@PLEL (OxP, R848, methylene blue) at different temperatures; C) *In vivo* sol-gel transformations of or/MB@PBS and or/MB@PLEL; D, E) storage modulus (G') and loss modulus (G”) of blank PLEL and or@PLEL with temperature. Reprinted with permission from ref (Wang et al., 2023). ©by *Meng Wang* et al.

In addition to loading chemotherapeutic agents, thermo-responsive hydrogels can simultaneously deliver immunotherapeutic agents to enhance the therapeutic efficacy in patients with peritoneal CRC metastases. Resiquimod (R848) is an immune response modifier, which, in combination with oxaliplatin, synergistically enhances dendritic cell maturation, promotes cytotoxic T-lymphocyte expansion, and induces macrophage differentiation into an antitumor phenotype (Zhang et al., 2023). PLEL hydrogel is a fluidizable solution state at room temperature, which allows the convenient loading and injection of drugs using this hydrogel. Following *in vivo* administration, a semi-solid hydrogel was formed at 37 °C. This could aid the sustained and gradual release of the drug over a 72-hour period, effectively prolonging the drug retention time. Wang et al. used a thermo-responsive and injectable PLEL hydrogel to aid the local deposition of oxaliplatin and R848 in the peritoneal cavity, which significantly suppressed ascites exudation and prolonged survival in a mouse peritoneal metastatic tumor model (Figure 4) (Wang et al., 2023). PEG hydrogels that remained stable after a sol-gel transition that can occur at body temperature also contributed to the synergistic effect of the molecularly targeted agents regorafenib and ICI in CRC treatment (Zhang et al., 2021).

### 6.2. pH-responsive smart hydrogels

The acidic microenvironment in the TME is the foundation of pH-responsive hydrogels for drug delivery in tumor therapy. Lactic acid and other acidic metabolites produced by rapidly-growing tumor cells establish a pH ranging from 5.8 to 7.2 in the TME and an even lower pH in the lysosomes or endosomes responsible for aiding drug release (Wang et al., 2020). Unlike that in normal tissues, pH-responsive hydrogels can be used in the TME for the targeted delivery of drugs at the tumor site. The primary components of a pH-responsive hydrogel typically include a polymer matrix and a functional group. The polymer matrix usually contains a natural or synthetic polymer, such as gelatin, chitosan, PEG, and PLA. Functional groups such as PAA and PMAA, which have numerous carboxyl groups, can be protonated or deprotonated under different pH environments. This alters the overall molecular charged property, which endows the hydrogel with responsiveness to pH changes (Xu et al., 2013). In the acidic environment of tumor tissues, acidic groups undergo dissociation or hydrolysis, which leads to structural changes in the hydrogel and release of the drug. This pH-responsive release mechanism allows targeted drug delivery, in which the drug is delivered precisely into the tumor tissue. This improves the therapeutic efficacy and reduces the damage to adjacent healthy tissue.

**Figure 5.**
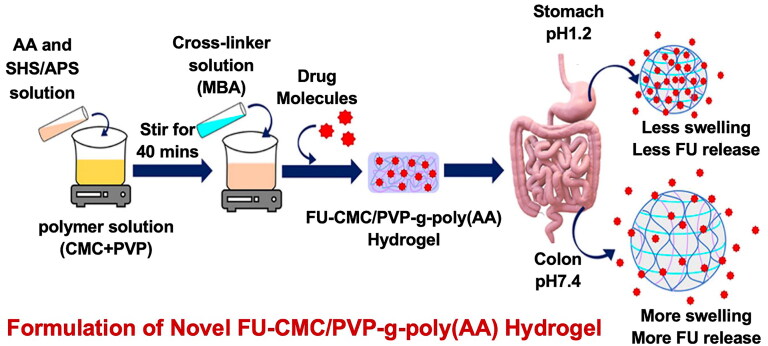
The pH-responsive mucosal adhesion hydrogel FU-CMC/PVP-g-poly(AA) undergoes swelling at colonic site pH, releasing more 5-Fu and serving as an ideal oral drug carrier. Reprinted with permission from ref (Hanan et al. 2024). ©by 2024 Elsevier B.V.

The pH-responsive hydrogels can facilitate oral drug delivery based on the pH in various parts of the GIT (Figure 5) (Hanan et al., 2024). Batool et al. delivered cytarabine in PMMA hydrogels and demonstrated that the drug platform showed more pronounced pH-induced degradation and drug release in the gut without causing excess ocular, dermal, and oral toxicity. This significantly prolonged the half-life of the drug as compared to that in commercially available oral solutions (Batool et al., 2022). Naringenin is a natural anticancer agent, but its low aqueous solubility and poor bioavailability considerably limit its clinical applications. Md et al. used pH-responsive bilayer nanohydrogels to help enhance naringenin solubility and facilitate its targeted colonic release for CRC treatment (Md et al., 2022). Abbasi et al. modified the surface of pH-responsive hydrogels with FA ligands to aid the oral delivery of hydrophobic dioxolane through the dual action of pH responsiveness and active targeting of FA ligands (Abbasi et al., 2023). pH-responsive hydrogels for oral administration can also help deliver *Agaricus bisporus* polysaccharides with NK cell-modulating effects (El-Deeb et al., 2022). Graphene quantum dots, as a preferred material for drug delivery and bioimaging, can be combined with pH-responsive CMC hydrogels as a cross-linking agent to impart fluorescence bioimaging capabilities (Rakhshaei et al., 2020).

Tumor tissues are considered more acidic than normal tissues, making pH-responsive hydrogels a powerful tool for differentiating between these tissues. pH-responsive hydrogels effectively reduce drug accumulation and systemic toxicity in normal tissues and enhance therapeutic efficacy by facilitating acidic tumor site-specific drug release.

### 6.3. Redox-responsive smart hydrogels

**Figure 6.**
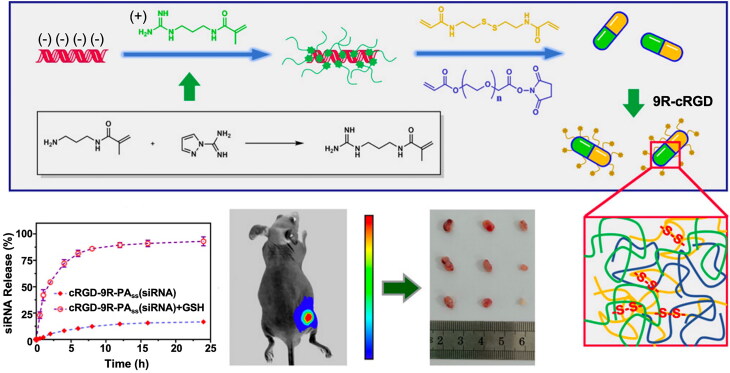
Redox-responsive polyacrylamide nanohydrogels facilitate delivery of siRNAs for the treatment of CRC. Functional groups such as the tumor-targeting peptide cRGD, the cell-penetrating and lysosomal escape peptide 9 R, and redox-responsive disulfide bonds were introduced into polyacrylamide nanohydrogels to facilitate delivery of STAT3 siRNA. Reprinted with permission from ref (Yu et al. 2024). ©by 2024, American chemical society.

Rapidly proliferating tumor cells with an active metabolism and a hypoxic TME contribute to the abnormally elevated levels of glutathione (GSH) in tumor tissues, especially in multidrug-resistant tumors, where cytoplasmic GSH levels are four times greater than those in healthy tissues. Redox-responsive hydrogels have chemical bonds that are susceptible to degradation at high GSH concentrations, such as sulfide bonds and disulfide bonds. The reducing groups present in hydrogels may undergo redox reactions in the presence of high concentrations of reducing substances, such as GSH, leading to fracture, de-crosslinking, or structural changes in the hydrogel. Redox-responsive hydrogels are designed to release drug-carrying substances by triggering a change in the hydrogel structure in the presence of high concentrations of reducing substances in tumor tissues. Yan et al. used a disulfide-bonded polymer hydrogel that undergoes hydrolysis at high GSH concentrations to aid the delivery of DOX for CRC treatment. This enhanced the CRC cell-eliminating ability by 5,000-fold compared to that of free DOX (Yan et al., 2010). GSH present at high concentrations in tumor tissues acts as an antioxidant to protect cells from oxidative damage, a property that significantly limits the therapeutic efficacy of PT and SDT. Chen et al. prepared a composite hydrogel that could modulate the tumor redox microenvironment. This comprised the acoustic sensitizer protoporphyrin IX and a GSH inhibitor, which could significantly inhibit the growth of xenograft tumors in a mouse model (Chen et al., 2022). Redox-responsive hydrogels can also be applied to help deliver siRNA to tumor cells to silence target genes. Yu et al. introduced functional groups such as tumor-targeting peptide cRGD, cell-penetrating and lysosomal escape peptide 9 R, and redox-responsive disulfide bonds into polyacrylamide nanohydrogels, which could successfully release STAT3 siRNA response into CT26, promoting STAT3 gene silencing and inhibiting cancer cell proliferation (Figure 6) (Yu et al., 2024).

As an important gas molecule, hydrogen sulfide (H_2_S) is considered to contribute to the development of several diseases and promote the proliferation of CRC cells and angiogenesis at tumor sites. Li et al. modified HA with sulfhydrylation to facilitate its attachment to the intestine via disulfide bonding at high GSH levels and loaded it with both *Thiobacillus nitrophilus* and comedoine. *T. nitrophilus* could oxidize sulfide in the anaerobic TME and continuously and effectively scavenge H_2_S. This bacterial hydrogel could effectively reduce the H_2_S level in the TME, normalize the tumor vasculature, increase the targeting and release of comedoine, and significantly inhibit the proliferation of colon cancer cells and angiogenesis at the tumor site (Li et al., 2023).

### 6.4. Light-responsive smart hydrogels

As an external stimulus, light can induce structural changes in the hydrogel network carrying the photosensitizer, leading to drug release. Meanwhile, a material with excellent photothermal conversion efficiency encapsulated in the hydrogel can convert light energy into heat energy in the presence of an external light stimulus, which triggers the necrosis or apoptosis of tumor cells (Yan et al., 2021). Besides, some photosensitizers can generate ROS under irradiation at specific wavelengths, initiating a photochemical reaction in the tumor cells and inducing cell damage and death (Rodrigues & Correia, 2022).

As a common photothermal agent, silver nanoparticles (AgNPs) can convert light energy into heat energy through the surface plasmon resonance effect under excitation with visible light, causing an increase in the temperature within the hydrogel. This promotes drug release and aids tumor treatment. Freitas et al. loaded curcumin and AgNPs with chitosan/chondroitin sulfate hydrogel and demonstrated that the hydrogel can significantly eliminate Caco-2 cells through metal-enhanced single-linear oxygen effect under visible light. This agent could be used as a fluorescent probe for diagnostic purposes (de Freitas et al., 2020). Iron oxide nanoparticles can induce CT26 cell death *in vitro* by photothermal effect under NIR excitation and have also been used in studies on drug-loaded therapy for CRC (Ji & Wang, 2023).

Identifying materials with high photothermal conversion efficiency is a critical aspect of preparing light-responsive hydrogels. Dopamine nanoparticles have a wide range of absorption and scattering spectra on the surface, which can exhibit absorption at a wide range of light wavelengths with high photothermal conversion efficiency. Moreover, dopamine nanoparticles have favorable biocompatibility and are facile to prepare and have thus attracted the attention of researchers (GhavamiNejad et al., 2016). Kim et al. prepared an HA-dopamine hydrogel loaded with PD-1 antibody to effectively combine immunotherapy and PT. This could significantly inhibit the growth of primary and metastatic tumors (Kim et al., 2023).

**Figure 7.**
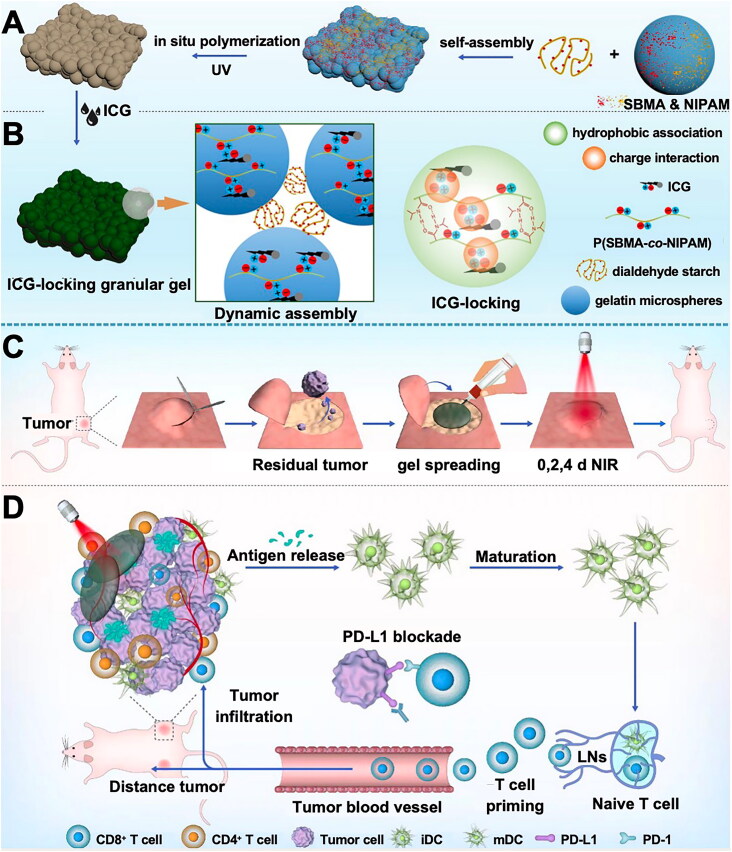
Tissue-adhesive hydrogels loaded with indocyanine green and ICI that mediate PTT combined with immunotherapy are effective in preventing CRC recurrence and metastasis after surgery. A) Illustration of granular hydrogel fabrication, B) structure, C) application, and D) therapy mechanism. Reprinted with permission from ref (Zeting et al. 2023). ©by *Yuan Zeting* et al.

Indocyanine green, as a PS with favorable bioavailability, is widely used in the development of light-responsive smart hydrogels. Zeting et al. prepared a granular hydrogel interpenetrated by gelatin and poly (sulfobetaine methacrylate-N-isopropylacrylamide), which could cause the water-soluble indocyanine green to adhere to the tumor site and prevent its rapid diffusion and clearance (Figure 7) (Zeting et al., 2023). Indocyanine green particulate hydrogel-mediated PTT can be used to eliminate residual and metastatic tumor cells after surgery and prevent long-term tumor recurrence.

Light-responsive hydrogels are gaining popularity owing to their unique ability to undergo photopolymerization *in situ*, highly controllable drug release, and synergistic therapeutic properties. The structure of hydrogels can also be modified to precisely control their ability to load and release drugs, enabling more effective treatment. In addition, combination therapies and novel photothermal conversion materials may be developed in the future, leading to a stronger role for hydrogels as DDS in tumor therapy.

### 6.5. Magnetic-responsive smart hydrogels

Magnetic response as an external stimulus for smart hydrogels is distinctly characterized by remote manipulation and magnetic resonance imaging. Magnetic-responsive hydrogels prepared by embedding magnetic nanomaterials into hydrogel networks exhibit significant advantages in biomedical applications owing to their rapid magnetic response, precise temporal and spatial control, and noninvasive remote actuation (Fragal et al., 2022). When subjected to an external magnetic field, magnetic hydrogels can be manipulated to achieve multiple response modes, such as movement, deformation, and heat production for therapeutic purposes without being limited by the depth of tissue penetration. The primary magnetic fields used to stimulate hydrogels include static magnetic fields (SMFs) and alternating magnetic fields (AMFs) (Li et al., 2021). SMFs can guide magnetic-responsive hydrogels to desired sites and aid the targeted release of drugs. AMFs cause magnetic materials embedded in thermosensitive hydrogels to absorb electromagnetic energy and convert it into thermal energy, inducing drug release and facilitating the treatment of tumors with PT.

**Figure 8.**
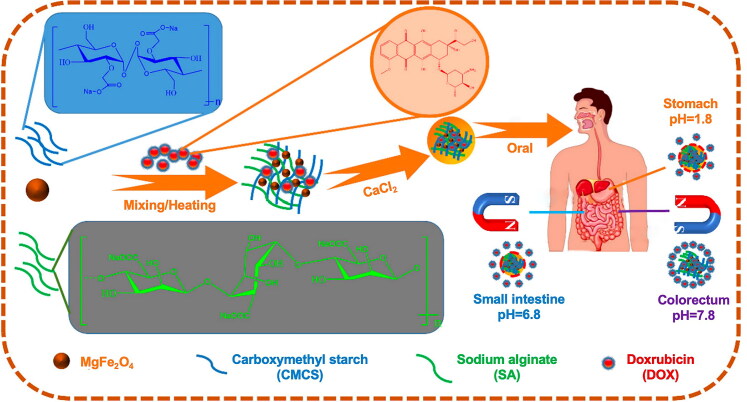
Magnetic-responsive hydrogel assists in oral delivery of DOX. The magnetic and pH dual-responsive drug platform CMCS-SA@MgFe2O4@Dox hydrogel could accelerate the release of DOX at different sites of the GIT under the influence of pH and external magnetic fields at the colonic site. Reprinted with permission from ref (Fang et al. 2022). ©by 2022 Elsevier B.V.

SMF enables the guidance of magnetic-responsive hydrogels to the desired site for targeted drug delivery. Amini Fazil et al. used chitosan/polyacrylic acid/Fe_3_O_4_ magnetic-responsive hydrogels loaded with 5-Fu to significantly enhance the drug dosage stability and facilitated the long-term controlled release of drug in the colon and rectum in the presence of SMF (Amini-Fazl et al., 2019). Similarly, Fang et al. prepared carboxymethyl starch/arginine hydrogels incorporating MgFe_2_O_4_ nanoparticles, which are both pH- and magnetically-responsive and could be used for the oral administration of DOX (Figure 8) (Fang et al., 2022). Drug release experiments revealed the excellent stability of this DDS in simulated gastric fluids. DOX was released stably and continuously in intestinal fluids. An SMF significantly accelerated drug release and exerted a strong toxic effect on the HCT116 human CRC cell line.

AMF performed excellently in inducing the drug release and structural deformation of thermo-responsive hydrogels (Sun et al., 2020). Chen et al. prepared injectable hydrogels composed of arachidonic acid-modified amphiphilic copolymers encapsulating Zn_0.4_Fe_2.6_O_4_ nanocubes with an efficient hysteresis loss mechanism and the iron-death inducer RSL3 (Chen et al., 2023). The system could achieve solution-gel transformation under AMF, accelerate the release of RLS3, enhance the antitumor efficacy of iron-induced cell death, and induce a magnetothermal therapeutic effect *via* the synthesis of ROS in large amounts, a synergistic strategy that achieved complete elimination of CT-26 tumors in mice.

As drug carriers with low toxicity and a high drug loading capacity, magnetic-responsive hydrogels can also be used for the precise monitoring of tumor conditions to achieve efficient imaging-guided tumor treatment. Wu et al. developed a novel chitosan superparamagnetic iron oxide nanocarrier loaded with indocyanine green and irinotecan, which enabled the fluorescence real-time monitoring of the efficiency of magnetically-targeted drug delivery to the lesion under an applied magnetic field (Wu et al., 2020). The drugs were effectively enriched at the tumor site under the applied magnetic field.

### 6.6. Enzyme-responsive smart hydrogels

Enzyme-responsive hydrogels normally contain sensitive moieties that can be hydrolyzed by enzymes that are expressed at high levels in tumor tissues, such as proteases and glycases. In case of exposure to these enzymes, the corresponding moiety recognizes and hydrolyzes the bonding sites in the hydrogel network, leading to the dissociation or alteration of the hydrogel structure. This facilitates the targeted release of the drug. Wang et al. prepared intestinal enzyme-responsive chitosan hydrogels to enhance the effects of the hydrophobic molecularly-targeted drug imatinib in CRC therapy. The hydrogel also delivered the osmotic facilitator sodium deoxycholate to promote drug penetration into tumor cells (Wang et al., 2022). They synthesized methacrylic anhydride-modified carboxymethyl chitosan hydrogels by amidation. Enzymatic drug release from the loaded imatinib hydrogel could be achieved using intestinal lysozymes to hydrolyze the glycosidic bonds on the sugar chain. Meanwhile, sodium deoxycholate loaded therein could help open the epithelial tight junctions and improve intestinal permeability.

Metalloproteinases (MMPs), which are endopeptidases capable of cleaving peptide bonds, are involved in several processes, such as tumor cell adhesion, proliferation, differentiation, migration, and cellular interactions. These serve as important targets for enzyme-responsive antitumor DDS. The specific peptide-bound amino acid-conjugated hydrogels sensitive to MMP activity are subjected to degradation and drug release in the presence of MMPs (Nultsch & Germershaus, 2018). Nazli et al. synthesized integrin-targeted and MMP-responsive PEG hydrogels for loading DOX. These hydrogels efficiently delivered DOX into tumor cells within 2 h (Nazli et al., 2014).

**Figure 9.**
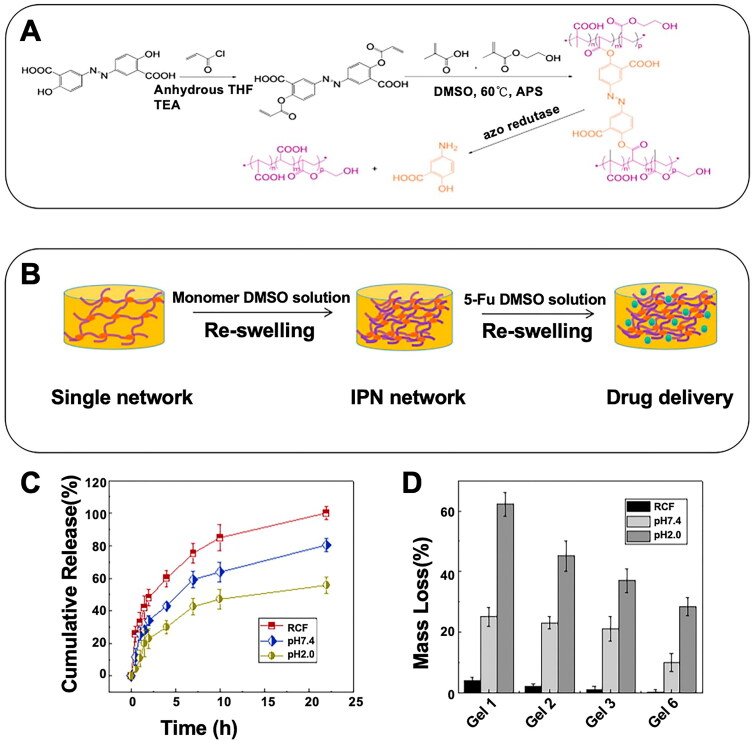
Enzyme-responsive hydrogels based on olsalazine derivatives to aid specific delivery of 5-Fu in the Colon. (A) Synthesis route for hydrogels; (B) Schematic design of drug-loaded hydrogels; (C) IPN network hydrogels released 5-Fu under different pH and rat colonic fluid conditions; (D) mass loss of azo hydrogels degraded for 24 h under three conditions. Reprinted with permission from ref (Ma et al. 2019). ©by 2019 Elsevier.

The unique flora of the colon and its production of azoreductases (Azored) has led researchers to investigate the role of azo-aromatic bonds, which can be degraded by Azored, in colon-specific DDS (Yeh et al., 1994). Azo-aromatic bonds are commonly used as a cross-linking agent in pH-responsive hydrogels, wherein they protect the drug from digestion by acidic gastric juices. Following the entry of the hydrogel into the intestine, the carboxyl groups present in the hydrogel network are ionized and subjected to colonic Azored degradation for drug release (Yeh et al., 1995). Through the combination of enzyme responsiveness and pH responsiveness, the drug is delivered to the target site with greater precision and released *via* a biodegradation mechanism. Ma et al. prepared a colon-specific 5-Fu hydrogel DDS using Azored (Figure 9) (Ma et al., 2019). They co-polymerized a hydrogel composed of hydroxyethyl methacrylate and methacrylic acid with acryloyl chloride-modified olsalazine as an azo cross-linking agent. The drug release profiles indicated that 5-Fu loaded in the azo-hydrogel was released more rapidly from rat colonic fluids. The presence of dextranase in the colon enables dextran hydrogels cross-linked with diisocyanates to deliver drugs in CRC.

### 6.7. Special-responsive smart hydrogels

SDT is an emerging tumor therapeutic method that serves biological tissues. It has two key effects: thermal and non-thermal. The thermal effect can convert acoustic energy into thermal energy, which increases the local temperature in tumor tissues to kill tumor cells (Gao et al., 2005). The non-thermal effect is associated with the minute bubbles generated by ultrasound vibration, which subsequently increase cell membrane permeability and excite acoustic sensitizers to produce ROS and induce the apoptosis of tumor cells (Manouras & Vamvakaki, 2017). Advantages such as deep penetration and easy visualization of ultrasound-responsive hydrogels make them a preferred DDS. These hydrogels may be suitable for both diagnostic and therapeutic applications of ultrasound. Chen et al. delivered the acoustic sensitizer protoporphyrin IX and a GSH synthesis inhibitor in an alginate hydrogel and performed SDT after local injection at the tumor site (Chen et al., 2022). The generated ROS induced apoptosis in tumor cells, whereas the GSH inhibitor improved the reducing TME, increased intracellular oxidative stress, and enhanced SDT efficacy. Ultrasound-responsive hydrogels can also be used to help enhance the efficacy of immunotherapy. Cao et al. reported the use of chitosan/alginate hydrogels delivering the acoustic sensitizers mesoporous manganese oxide and ICI. These were found to directly kill tumor cells, reverse the immunosuppressive TME, and enhance systemic antitumor immunity in an animal model when delivered *via* the oral route (Cao et al., 2022).

The ROS level is typically elevated in tumor tissues. Potential reasons for this include the abnormal metabolic state of the tumor tissue, hypoxia, and an acidic TME. These tend to result in abnormally elevated levels of intracellular oxidative stress and metabolically vigorous tumor cells, which cause aberrations in the mitochondrial electron transport chain (Cui et al., 2018). ROS-sensitive groups, such as thioether and peroxide bonds, are subject to redox reactions when stimulated by ROS. This leads to the fracture, cross-linking, or dissolution of the hydrogel structure and facilitates the targeted release of the drug in the tumor tissue. Li et al. developed a dual thermal- and ROS-responsive composite hydrogel using ROS-responsive thioredoxin as a cross-linker for delivering regorafenib and transforming growth factor-β (TGF-β) inhibitors (Li et al., 2022). Regorafenib was preferentially released under thermal stimulation after local injection at the tumor site and increased the intracellular ROS levels, subsequently triggering the release of ROS-responsive TGF-β inhibitors. The sequential release of regorafenib and TGF-β inhibitors promoted the antitumor immune response by reprogramming immune cells, which effectively inhibited tumor growth and metastasis. ROS-responsive smart hydrogels may also be available to aid the treatment of CRC peritoneal metastases, bridging low-dose fractionated radiotherapy and ICB therapy. Radiation therapy induces apoptosis in tumor cells by direct DNA destruction as well as by increasing the ROS-induced oxidative load. These can act as internal stimuli to induce the deformation of hydrogels containing borate bonds and the release of drugs. Liu et al. designed an *in situ*-forming hydrogel based on phenylboronic acid polymer and tannic acid loaded with aPDL1 and aCD7 (Liu et al., 2023). The hydrogel could form locally and adhere to the peritoneum after intraperitoneal injection. The radiotherapy-induced generation of ROS could rupture the borate esters in the hydrogel to release antibodies, triggering an obvious antitumor response.

Electro-responsive hydrogels with functional groups, such as charged ions or electrode-active groups, can cause structural changes in the hydrogel under an external electric field. This allows the targeted release of drugs and the precise treatment of tumor tissues. Bakalova et al. investigated an electrically-assisted delivery hydrogel loaded with QD^705^ and manganese that could effectively promote the delivery of nanohydrogels in tumor tissues in the presence of an electric field, as observed by fluorescence imaging and nuclear magnetic resonance imaging (Bakalova et al., 2016). Moreover, the increased permeability of blood vessels in tumor tissues in the electroporated region can improve drug penetratation through the barrier and enhance the effects of the drug.

Different parts of the digestive tract have different flora compositions, and the intestinal flora is closely related to a multitude of diseases, which is the basis for the action of bacteria-responsive hydrogels. The unique hypoxic environment of the TME aids the targeting action of anaerobic bacteria. Hydrogels driven by anaerobic bacteria can selectively grow in tumors and may be used as novel biocarriers for drug delivery and tumor therapy. *Bifidobacterium infantis* (BI) is a common intestinal flora that can be easily cultured and surface-modified, has favorable biosafety, and can function as a controlled DDS targeting CRC (Mavrich et al., 2018; Lou et al., 2021). Given that BI can selectively grow in the hypoxic TME of CRC, Zhang et al. prepared a hybrid drug delivery system loaded with endothelin and DOX that downregulated VEGF and exerted an anti-angiogenic effect while eliminating tumor cells, thus exhibiting excellent antitumor effects (Zhang et al., 2022). *Thiobacillus nitrophilus*, which can oxidize sulfide in an anaerobic TME and continuously and efficiently scavenge H_2_S, is also being investigated for its role in drug-carrying therapies for CRC (Liechty et al., 2019). Intestinal bacteria may also be related to chemotherapy resistance in CRC. Recent studies support that the specific intestinal microorganism *Fusobacterium nucleatum* (*Fn*) can promote CRC chemotherapy resistance by targeting Toll-like receptors (TLR4 and MYD88) and microRNAs to regulate autophagy (Yan et al., 2022). Wu et al. developed a supramolecular galactosamine-derived nanoplatform that can block the interaction between *Fn* and host cells, thereby enhancing the chemotherapy effect of CRC (Wu et al., 2024).

### 6.8. Multi-responsive hydrogel-based controlled drug release systems

Multi-responsive hydrogels respond to various internal or external stimuli to achieve sustained drug release during treatment, increasing the concentration of the drug within tumor tissues and enhancing its therapeutic effect. Multi-responsive hydrogels facilitate drug enrichment within tumor tissues and reduce the impact on healthy tissues and the side effects of the treatment. The changes in the physical properties of the hydrogel can also be monitored to realize real-time monitoring, regulate the treatment process, and improve the effectiveness and safety of the treatment, which is the focus of current research. RNA interference (RNAi) gene therapy is a therapeutic approach in which tumors are treated by mediating the RNA interference pathway to inhibit the expression or function of specific genes. The major issue with its wide clinical application is the efficient delivery of RNAi to tumor cells. B. Liecht et al. prepared pH- and redox-multi-responsive hydrogels to assist the delivery of functional siRNAs (Liechty et al., 2019). They prepared a dual-responsive hydrogel by introducing disulfide as a cross-linker in a pH-responsive 2-(diethylaminoethyl) methacrylate hydrogel. The hydrogel could form synergistic electrostatic interactions with polyanionic siRNA and degrade in the redox TME, releasing siRNA and inducing gene knockdown for therapeutic purposes.

Multi-responsive hydrogels can respond to unique stimuli in the TME, prompting targeted drug release at the tumor site and reducing damage to healthy tissues. Xie et al. prepared a pH- and redox-responsive chitosan DDS by co-precipitation to realize dual-responsive DOX-triggered release (Xie & Liu, 2020). The DDS was experimentally demonstrated to have a drug loading capacity greater than 36%, and the excellent pH and redox dual response triggered a cumulative DOX release greater than 85% in a simulated TME. Meanwhile, the drug leakage in simulated normal physiological media was minimal. Liang et al. combined pH-responsive chitosan/oxidized dextran hydrogels with nanoflowers attracted to MoS_2_ via hydrogen bonding and electrostatic attraction to aid the local release of 5-Fu and methotrexate (Liang et al., 2023). Methotrexate was released from the drug carrier at a pH of 7.4, and MoS_2_, as an excellent photothermite, could generate high levels of heat under NIR irradiation and release the encapsulated 5-Fu. The sequential mode of delivery significantly enhanced the therapeutic effect on CRC.

Thermo-responsive hydrogels combined with photosensitizers can respond to both internal and external thermal stimuli, providing better assistance in the targeted release of antitumor drugs and exerting antitumor effects. Zheng et al. used a thermo-responsive chitosan hydrogel wrapped with the photothermal materials MoS_2_/Bi_2_S_3_-PEG (MBP) and DOX to combine local PTT and chemotherapy (Zheng et al., 2019). This gel system could release DOX under NIR excitation after injection into the tumor site for efficient targeted therapy.

## 7. Challenges and varied perspectives on hydrogels applied to CRC

Achieving efficient CRC treatment is a major clinical challenge. Efficient drug carriers can facilitate the precise targeting of chemotherapeutic and immunological drugs to the tumor site and serve as a useful strategy in tumor therapy research. In recent years, consistent progress in research on various novel polymeric materials and nanotechnology has led us to innovative ideas on drug carriers in tumor therapy. Hydrogel, as a three-dimensional network material, has attracted the attention of researchers for its low toxicity, excellent biocompatibility, reversible swelling, and biodegradability. Additionally, smart hydrogels that can respond to internal and external stimuli have become the focus of research.

There are many advantages of hydrogels as emerging oncology drug carriers. Their favorable biocompatibility makes them degrade naturally in physiological conditions without causing obvious immune reactions or toxic side effects. The network-like structure allows hydrogels to carry a large number of drug molecules, enabling the efficient treatment of tumor tissues and reducing the number of drug administrations. The easy-to-modify surface helps researchers achieve targeted drug release by adjusting the structural properties and porosity of the hydrogel or introducing specific active chemical groups. Hydrogels also enable the slow release and targeted delivery of drugs, thus ensuring that the drug concentration in tumor tissues is maintained within the effective therapeutic range. Over the past decades, several successful studies have been conducted on the applications of hydrogels as drug delivery vehicles in tumor therapy. *In situ* hydrogels can be used as carriers for drugs with poor complex solubility and low stability as well as local reservoirs for the sustained delivery of multiple drugs. Hydrogels also have large surface area and can respond to various stimuli efficiently. Smart-responsive hydrogels can perceive changes in internal and external environmental stimuli (such as temperature, pH, redox, light, magnetic field, and enzyme), and respond accordingly to control the directional release of drugs and ensure a high concentration of drugs in the tumor site without causing unnecessary adverse reactions. This in turn effectively resolves common challenges encountered during clinical treatment.

However, most research on hydrogel drug-carrying therapies remains limited to the cellular or animal level, with several obstacles in the translation of experimental findings to clinical applications: (a) Drug stability: appropriate embedding or encapsulation techniques are necessary to protect the drug and prolong its stability in the hydrogel to avoid degradation or inactivation during storage, delivery, and release. Moreover, *in vivo* drug release is affected by multiple factors, making accurate pharmacokinetic monitoring difficult. Also, selecting suitable methods to achieve the precise control of drug loading and release rate is a major requirement in clinical application. (b) Local environment of the tumor tissue: the high pressure and imperfect vascular system of the TME may affect the penetration and distribution of the hydrogel, limiting the efficacy of the drug. (c) Batch preparation and preservation: the transition from experimental research to clinical application is challenged by the large-scale reproducible preparation and screening of hydrogel drug carriers. (d) Personalized drug carriers: with continuous progress in tumor treatment research, personalized treatment protocols are becoming increasingly popular. This also necessitates precise control of the properties of DDS to satisfy the needs of different patients. (e) Biocompatibility and safety: drug carriers applied in tumor therapy must have excellent biocompatibility and safety to avoid adverse reactions and side effects. However, in the current study, it is found that hydrogels, as drug carriers, have some unavoidable side effects in the process of treating tumors. For example, injecting hydrogel will inevitably cause slight redness and pain at the injection site. The emergence of microneedle patch hydrogel has well solved this problem (Cheng et al., 2024). While the short-term biocompatibility of various materials can be evaluated in animal models, their long-term biocompatibility cannot be confirmed. This requires researchers to consider all processes that the materials can influence in a comprehensive manner. In terms of long-term safety, Karsh et al. implanted absorbable hydrogel spacers into patients to help reduce the collateral damage to surrounding tissues during radiotherapy for prostate cancer (Karsh et al., 2018). Through a follow-up of subjects for up to 3 years, it was found that the application of prostate-rectal hydrogel spacers can significantly reduce the rectal radiation dose, reduce rectal toxicity in the long term, and improve intestinal, urinary, and sexual quality of life. The OLYMPUS clinical trial selected patients with primary or recurrent urothelial carcinoma and administered instillation therapy with UGN-101 (a mitomycin-containing reverse thermal gel). The experiment proves that UGN-101 provides a kidney-sparing treatment option for patients with low-grade diseases (Matin et al., 2022). However, an increase in the number of instillations may lead to an increase in adverse events in the urinary system, the most common of which is ureteral stenosis.

Owing to these challenges, researchers are required to continuously improve material design and preparation techniques and optimize drug loading and release strategies to enhance the efficacy and safety of hydrogels for drug delivery in CRC therapies. The following aspects can be considered: (a) Optimize material design. Select hydrogel materials with good biocompatibility, such as natural polymer materials (such as chitosan, sodium alginate, *etc.*) or synthetic polymer materials (such as PEG, PAA, *etc.*). The biocompatibility of materials can be improved by surface modification, such as grafting bioactive molecules or coatings. (b) Precise control of drug release: Adopt advanced preparation technologies such as micro-nano technology and 3D printing technology to precisely control the structure and drug loading capacity of hydrogels and achieve stable drug release. (c) Combine multiple drug delivery strategies, such as jointly using nanoparticles, liposomes, etc., to improve the drug loading efficiency and stability and reduce the uncertainty of drug release. (d) Reasonably select degradable materials: Choose degradable hydrogel materials and control their degradation rates. By adjusting parameters such as the chemical structure and crosslinking degree of materials, timely degradation of hydrogels in the body can be achieved to avoid the accumulation of degradation products. (e) Improve implantation technology and safety: Optimize the implantation method of hydrogels. Adopt minimally invasive surgeries or local injections and other methods to reduce the risk of damage and infection to surrounding tissues. Strengthen post-implantation monitoring and management. Conduct imaging examinations and biological detections regularly to detect and deal with possible complications in time. In conclusion, hydrogel materials have broad application prospects in drug delivery for colorectal cancer tumors, but it is also necessary to fully recognize the possible side effects and long-term safety issues. By optimizing material design, precisely controlling drug release, reasonably selecting degradable materials, and improving implantation technology, these risks can be minimized and a more safe and effective strategy can be provided for the treatment of CRC.
